# Internal Representations of Temporal Statistics and Feedback Calibrate Motor-Sensory Interval Timing

**DOI:** 10.1371/journal.pcbi.1002771

**Published:** 2012-11-29

**Authors:** Luigi Acerbi, Daniel M. Wolpert, Sethu Vijayakumar

**Affiliations:** 1 Institute of Perception, Action and Behaviour, School of Informatics, University of Edinburgh, Edinburgh, United Kingdom; 2 Doctoral Training Centre in Neuroinformatics and Computational Neuroscience, School of Informatics, University of Edinburgh, Edinburgh, United Kingdom; 3 Computational and Biological Learning Lab, Department of Engineering, University of Cambridge, Cambridge, United Kingdom; New York University, United States of America

## Abstract

Humans have been shown to adapt to the temporal statistics of timing tasks so as to optimize the accuracy of their responses, in agreement with the predictions of Bayesian integration. This suggests that they build an internal representation of both the experimentally imposed distribution of time intervals (the prior) and of the error (the loss function). The responses of a Bayesian ideal observer depend crucially on these internal representations, which have only been previously studied for simple distributions. To study the nature of these representations we asked subjects to reproduce time intervals drawn from underlying temporal distributions of varying complexity, from uniform to highly skewed or bimodal while also varying the error mapping that determined the performance feedback. Interval reproduction times were affected by both the distribution and feedback, in good agreement with a performance-optimizing Bayesian observer and actor model. Bayesian model comparison highlighted that subjects were integrating the provided feedback and represented the experimental distribution with a smoothed approximation. A nonparametric reconstruction of the subjective priors from the data shows that they are generally in agreement with the true distributions up to third-order moments, but with systematically heavier tails. In particular, higher-order statistical features (kurtosis, multimodality) seem much harder to acquire. Our findings suggest that humans have only minor constraints on learning lower-order statistical properties of unimodal (including peaked and skewed) distributions of time intervals under the guidance of corrective feedback, and that their behavior is well explained by Bayesian decision theory.

## Author Summary

Human performance in a timing task depends on the context of recently experienced time intervals. In fact, people may use prior experience to improve their timing performance. Given the relevance of time for both sensing and acting in the world, how humans learn and represent temporal information is a fundamental question in neuroscience. Here, we ask subjects to reproduce the duration of time intervals drawn from different distributions (different temporal contexts). We build a set of models of how people might behave in such a timing task, depending on how they are representing the temporal context. Comparison between models and data allows us to establish that in general subjects are integrating task-relevant temporal information with the provided error feedback to enhance their timing performance. Analysis of the subjects' responses allows us to reconstruct their internal representation of the temporal context, and we compare it with the true distribution. We find that with the help of corrective feedback humans can learn good approximations of unimodal distributions of time intervals used in the experiment, even for skewed distributions of durations; on the other hand, under similar conditions, we find that multimodal distributions of timing intervals are much harder to acquire.

## Introduction

The ability to estimate motor-sensory time intervals in the subsecond range and react accordingly is fundamental in many behaviorally relevant circumstances [1], such as dodging a blow or assessing causality (‘was it me producing that noise?’). Since sensing of time intervals is inherently noisy [2], it is typically advantageous to enhance time estimates with previous knowledge of the temporal context. It has been shown in various timing experiments that humans can take into account some relevant temporal statistics of a task according to Bayesian decision theory, such as in sensorimotor coincidence timing [3], tactile simultaneity judgements [4], planning movement duration [5] and time interval estimation [6]–[8].

**Figure 1:**
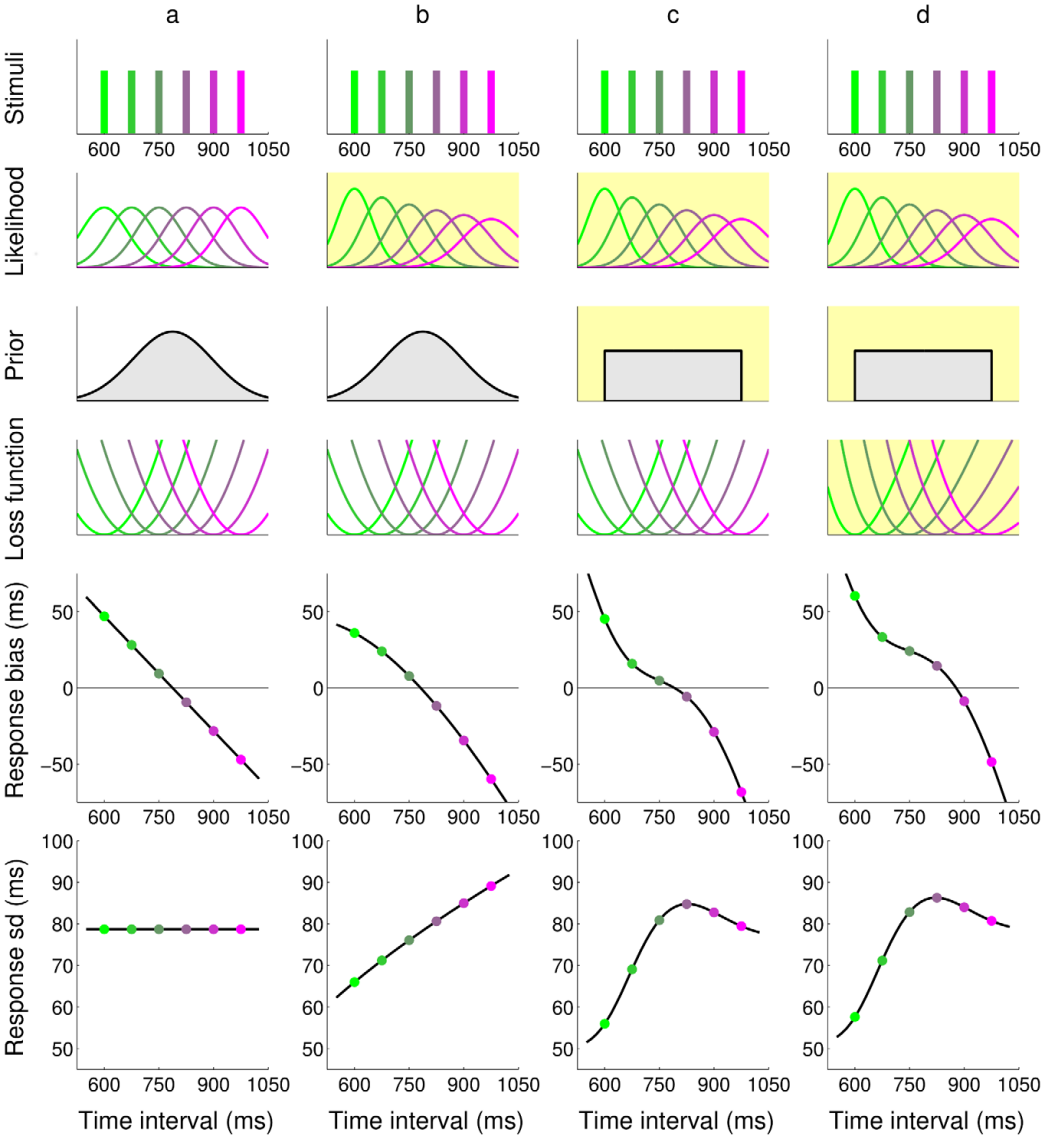
Comparison of response profiles for different ideal observers in the timing task. The responses of four different ideal observers (*columns* **a–d**) to a discrete set of possible stimuli durations are shown (*top row*); for visualization purpose, each stimulus duration in this plot is associated with a specific color. The behavior crucially depends on the combination of the modelled observer's temporal sensorimotor noise (likelihood), prior expectations and loss function (*rows* 2–4); see Figure 2 bottom for a description of the observer model. For instance, the observer's sensorimotor variability could be constant across all time intervals (column a) or grow linearly in the interval, according to the ‘scalar’ property of interval timing (column b–d). An observer could be approximating the true, discrete distribution of intervals as a Gaussian (columns a–b) or with a uniform distribution (columns c–d). Moreover, the observer could be minimizing a typical quadratic loss function (columns a–c) or a skewed cost imposed through an external source of feedback (column d). Yellow shading highlights the changes of each model (column) from model (**a**). All changes to the observer's model components considerably affect the statistics of the predicted responses, summarized by response bias, i.e. average difference between the response and true stimulus duration, and standard deviation (*bottom two rows*). For instance, all models predict a central tendency in the response (that is, a bias that shifts responses approximately towards the center of the interval range), but bias profiles show characteristic differences between models.

Most of these studies [3], [4], [6], [8] exposed the participants to time intervals whose duration followed some simple distribution (e.g. a Gaussian or a uniform distribution), and then assumed that the subjects' internal representation of it corresponded to the experimental distribution. As a more realistic working hypothesis, we can expect the observers to have acquired, after training, an internal representation of the statistics of the temporal intervals which is an approximation of the true, objective experimental distribution. It can be argued that this approximation in most cases would be ‘similar enough’ to the true distribution, so that in practice the distinction between subjective and objective distribution is an unnecessary complication. This is not exact though, first of all because it is unknown whether the similarity assumption would hold for complex temporal distributions, and secondly because the specific form of the approximation can lead to observable differences in behavior even for simple cases (see Figure 1).

We propose that understanding how humans learn and approximate temporal statistics in a given context can help explaining observed temporal biases and illusions [9]. Previous studies have shown that human observers exhibit specific idiosyncrasies in judging simultaneity and temporal order of stimuli after repeated exposure to a specific inter-stimulus lag (temporal recalibration) [4], [10], [11], in encoding certain kinds of temporal distributions in the subsecond range [12] or in estimating durations of very rare stimuli (oddballs) [13], so it is worth asking whether people are able to acquire an internal representation of complex (e.g. very peaked, bimodal) distributions of inter-stimulus intervals in the first place, and what are their limitations.

Bayesian decision theory (BDT) provides a neat and successful framework for representing the internal beliefs of an ideal observer in terms of a (subjective) prior distribution, and it gives a normative account on how the ideal observer should take action [14]. A large number of behavioral studies are consistent with a Bayesian interpretation [15]–[17] and some results suggest that human subjects build internal representations of priors and likelihoods [15], [18], [19] or likelihood and loss functions [20]. We therefore adopted BDT as a framework to infer the subjects' acquired beliefs about the experimental distributions. However, the behavior of a Bayesian ideal observer depends crucially not only on the prior, but also on the likelihoods and the loss function, with an underlying degeneracy, i.e. distinct combinations of distributions can lead to the same empirical behavior [21]. It follows that a proper analysis of the internal representations cannot be separated from an appropriate modelling of the likelihoods and the loss function as well.

With this in mind, we analyzed the timing responses of human observers for progressively more complex temporal distributions of durations in a motor-sensory time interval reproduction task. We provided performance feedback (also known as ‘knowledge of results’, or KR) on a trial-by-trial basis, which constrained the loss function, speeded up learning and allowed the subjects to adjust their behavior, therefore providing an upper bound on human performance [22], [23]. We carried out a full Bayesian model comparison analysis among a discrete set of candidate likelihoods, priors and loss functions in order to find the observer model most supported by the data, characterizing the behavior of each individual subject across multiple conditions. Having inferred the form of the likelihoods and loss functions for each subject, we could then perform a nonparametric reconstruction [24] of what the subjects' prior distributions would look like under the assumptions of our framework and we compared them with the experimental distributions. The inferred priors suggest that people learn smoothed approximations of the experimental distributions which take into account not only mean and variance but also higher-order statistics, although some complex features (kurtosis, bimodality) seem to deviate systematically from those of the experimental distribution.

## Results

**Figure 2:**
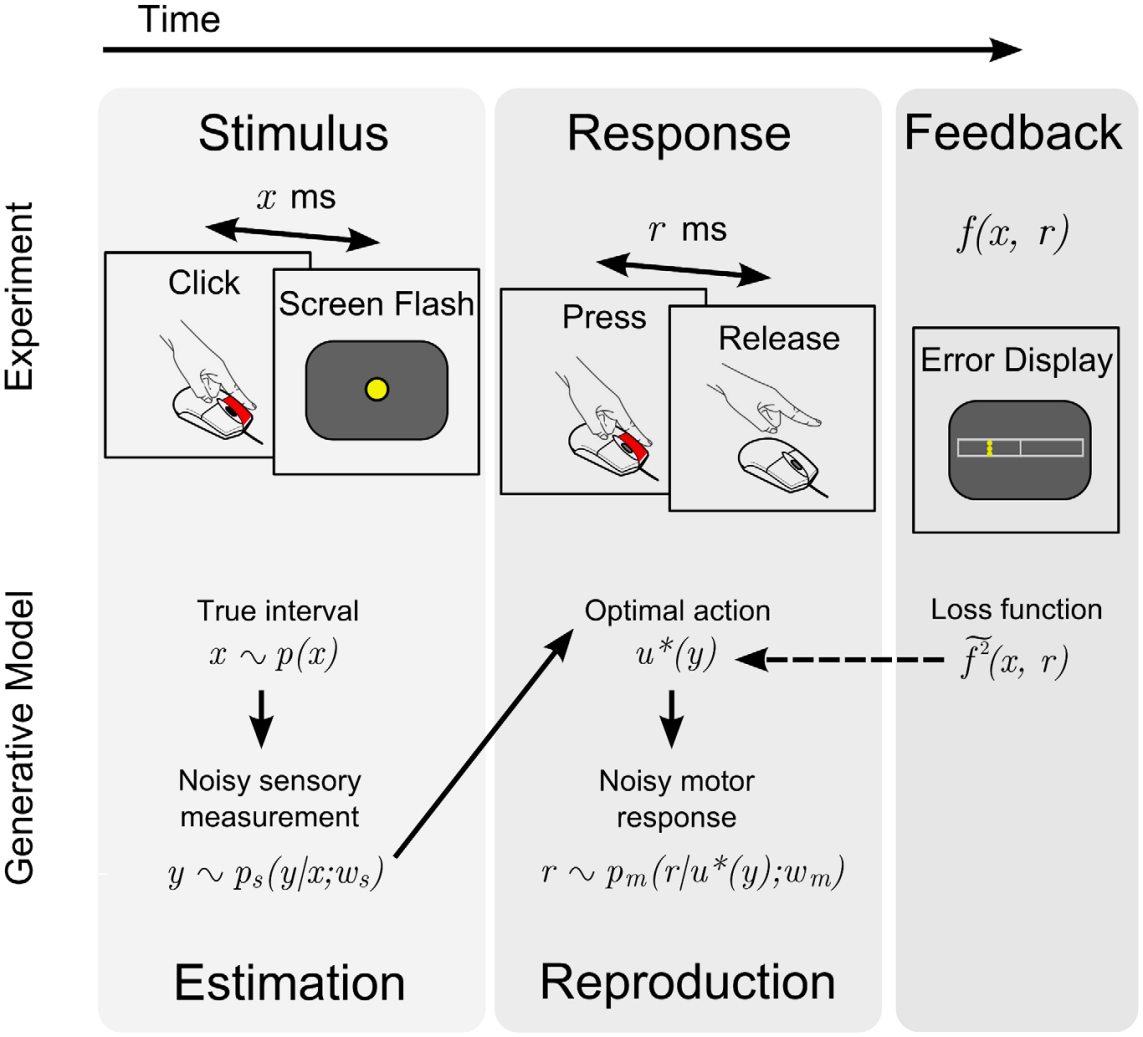
Time interval reproduction task and generative model. *Top:* Outline of a trial. Participants clicked on a mouse button and a yellow dot was flashed 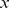 ms later at the center of the screen, with 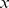 drawn from a block-dependent distribution (estimation phase). The subject then pressed the mouse button for a matching duration of 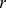 ms (reproduction phase). Performance feedback was then displayed according to an error map 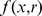. *Bottom:* Generative model for the time interval reproduction task. The interval 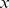 is drawn from the probability distribution 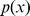 (the *objective distribution*). The stimulus induces in the observer the noisy sensory measurement 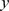 with conditional probability density 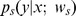 (the *sensory likelihood*), with 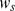 a sensory variability parameter. The action 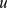 subsequently taken by the ideal observer is assumed to be the ‘optimal’ action 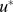 that minimizes the subjectively expected loss (Eq. 1); 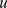 is therefore a deterministic function of 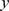, 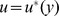. The subjectively expected loss depends on terms such as the prior 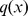 and the loss function (squared subjective error map 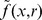), which do not necessarily match their objective counterparts. The chosen action is then corrupted by motor noise, producing the observed response 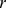 with conditional probability density 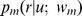 (the *motor likelihood*), where 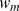 is a motor variability parameter.

Subjects took part in a time interval reproduction task with performance feedback (trial structure depicted in Figure 2 top; see Methods for full details). On each trial subjects clicked a mouse button and, after a time interval (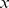 ms) that could vary from trial-to-trial, saw a yellow dot flash on the screen. They were then required to hold down the mouse button to reproduce the perceived interval between the original click and the flash. The duration of this mouse press constituted their response (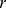 ms) for that trial. Subjects received visual feedback on their performance, with an error bar that was displayed either to the left or right of a central zero-error line, depending on whether their response was shorter or longer than the true interval duration. In different experimental blocks we varied both the statistical distribution of the intervals, 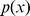, and the nature of the performance feedback, i.e. mapping between the interval/response pair and the error display, 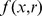, relative to the zero-error line. For each experimental block, subjects first performed training sessions until their performance was stable (around 500 to 1500 trials), followed by two test sessions (about 500 trials per session). Testing with a block was completed before starting a new one.

Different groups of subjects took part in five experiments, whose setup details are summarized in Table 1 (see also Methods). In brief, Experiment 1 represented a basic test for the experimental paradigm and modelling framework with simple (Uniform) distributions over different ranges. Experiment 2 compared subjects' responses in a simple condition (Uniform) vs a complex one (Peaked, one interval was over-represented), over the same range of intervals. Experiment 3 verified the effect of feedback on subjects' responses by imposing a different error mapping 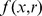. Experiment 4 tested subjects in a more extreme version of the Peaked distribution. Experiment 5 verified the limits of subjects' capability of learning with bimodal distributions of intervals.

**Table 1:** Summary of experimental layout for all experiments.

Experiment	Subjects	Interval range	Distribution	Peak probability	Feedback
1	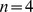	Short	Uniform	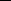	Skewed
		Long	Uniform	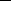	
2	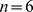	Medium	Uniform	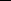	Skewed
		Medium	Peaked	7/12	
3	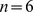	Medium	Uniform	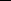	Standard
4	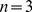	Medium	High-Peaked	19/24	Standard
5 a	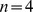	Medium	Bimodal	1/3 and 1/3	Standard
5 b	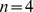	Wide	Wide-Bimodal	See text	Standard

Each line represents an experimental block, which are grouped by experiment; subjects in Experiment 1 and 2 took part in two blocks, whereas in Experiment 5 two distinct groups of subjects took part in the two blocks. For each block, the table reports number of subjects (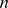), interval ranges, type of distribution, probability of the ‘peak’ (i.e. most likely) intervals and shape of performance feedback. Tested ranges were Short (450–825 ms), Medium (600–975 ms), Long (750–1125 ms) and Wide (450–1125 ms), each covered by 6 intervals (10 for the Wide block) separated by 75 ms steps. Distributions of intervals were Uniform (1/6 probability per interval), Peaked/High-peaked (the ‘peak’ interval at 675 ms appeared with higher probability than non-peak stimuli, which were equiprobable), Bimodal (intervals at 600 and 975 ms appeared with higher probability) and Wide-Bimodal (intervals at 450–600 ms and 975–1125 ms appeared with higher probability). The Skewed feedback takes the form 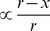 whereas the Standard feedback 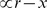, where 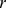 is the reproduced duration and 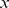 is the target interval in a trial.

We first present the results of the first two experiments in a qualitative manner, and then describe a quantitative model. Results of the other three experiments that test specific aspects of the model or more complex distributions are presented thereafter.

### Experiment 1: Uniform distributions over different ranges

In the first experiment the distribution of time intervals consisted of a set of six equally spaced discrete times with equal probability according to either a Short Uniform (450–825 ms) or Long Uniform (750–1125 ms) distribution. The order of these blocks was randomized across subjects. The feedback followed a Skewed error mapping 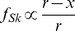. The ‘artificial’ response-dependent asymmetry in the Skewed mapping was chosen to test whether participants would integrate the provided feedback error into their decision process, as opposed to other possibly more natural forms of error, such as the Standard error 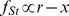 or the Fractional error 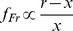 (see later, Bayesian model comparison).

**Figure 3:**
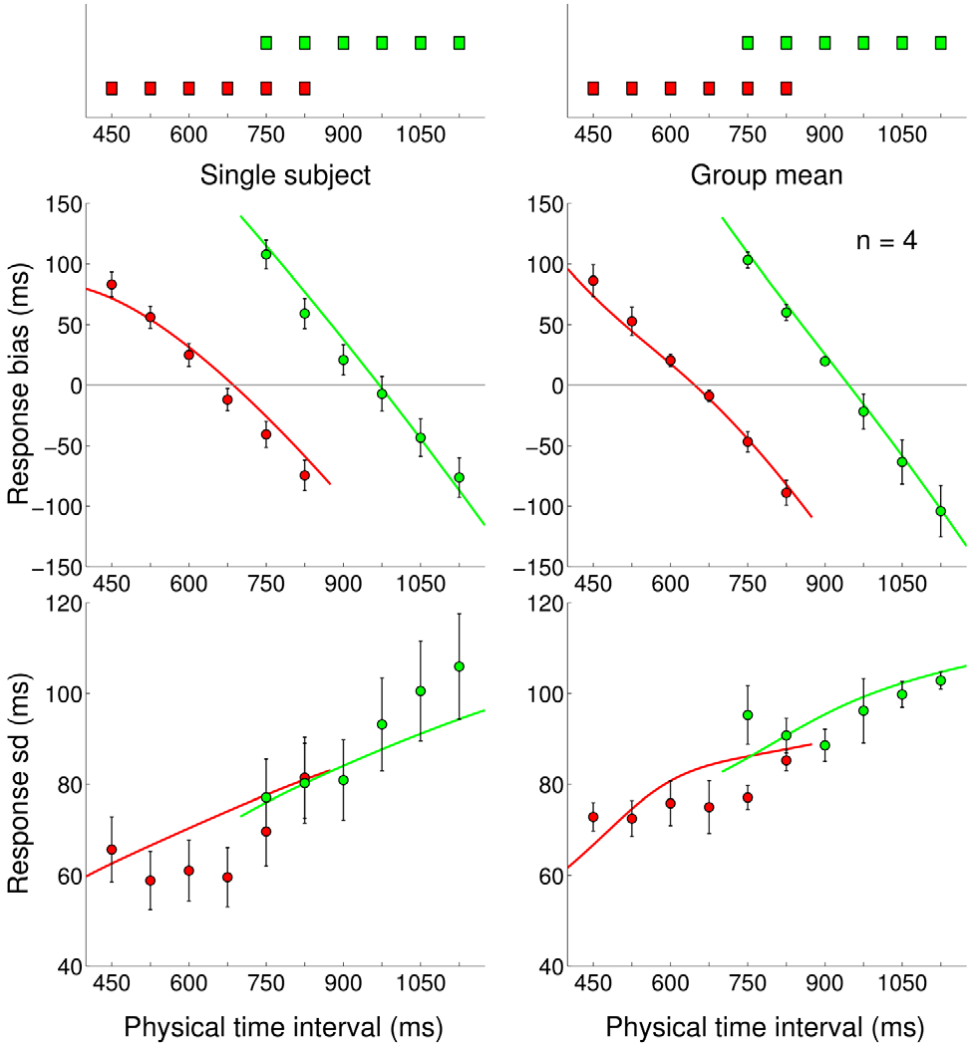
Experiment 1: Short Uniform and Long Uniform blocks. *Very top:* Experimental distributions for Short Uniform (red) and Long Uniform (green) blocks, repeated on top of both columns. *Left column:* Mean response bias (average difference between the response and true interval duration, top) and standard deviation of the response (bottom) for a representative subject in both blocks (red: Short Uniform; green: Long Uniform). Error bars denote s.e.m. Continuous lines represent the Bayesian model ‘fit’ obtained averaging the predictions of the most supported models (Bayesian model averaging). *Right column:* Mean response bias (top) and standard deviation of the response (bottom) across subjects in both blocks (mean 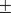 s.e.m. across subjects). Continuous lines represent the Bayesian model ‘fit’ obtained averaging the predictions of the most supported models across subjects.

We examined the mean bias in the response (mean reproduction interval minus actual interval, 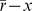, also termed ‘constant error’ in the psychophysical literature), as a function of the actual interval (Figure 3 top). Subjects' responses showed a regression to the mean consistent with a Bayesian process that integrates the prior with sensory evidence [4], [6], [8], [15]. That is, little bias was seen for intervals that matched the mean of the prior (637.5 ms for Short Uniform, red points, and 937.5 ms for Long Uniform, green points). However, at other intervals a bias was seen towards the mean interval of that experimental block, with subjects reporting intervals longer than the mean as shorter than they really were and conversely intervals shorter than the mean as being longer than they really were. Moreover, this bias increased almost linearly with the difference between the mean interval and the actual interval. Qualitatively, this bias profile is consistent with most reasonable hypotheses for the prior, likelihoods and loss functions of an ideal Bayesian observer (even though details may differ).

The standard deviation of the response (Figure 3 bottom) showed a roughly linear increase with interval duration, in agreement with the ‘scalar property’ of interval timing [25], according to which the variability in a timing task grows in proportion to the interval duration.

These results qualitatively suggest that the temporal context influences subjects' performance in the motor-sensory timing task in a way which may be compatible with a Bayesian interpretation, and in agreement with previous work which considered purely sensory intervals and uniform distributions [6], [8], [26].

### Experiment 2: Uniform and Peaked distributions on the same range

As in the first experiment six different equally-spaced intervals were used, with two different distributions. However, in this experiment both blocks had the same range of intervals (Medium: 600–975 ms). In one block (Medium Peaked) one of the intervals (termed the ‘peak’) occurred more frequently than the other 5 intervals, that were equiprobable. That is, the 675 ms interval occurred with 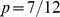 with the other 5 intervals occurring each with 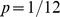. In the other block (Medium Uniform) the 6 intervals were equiprobable. The feedback gain for both blocks was again the Skewed error map 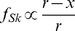.

**Figure 4:**
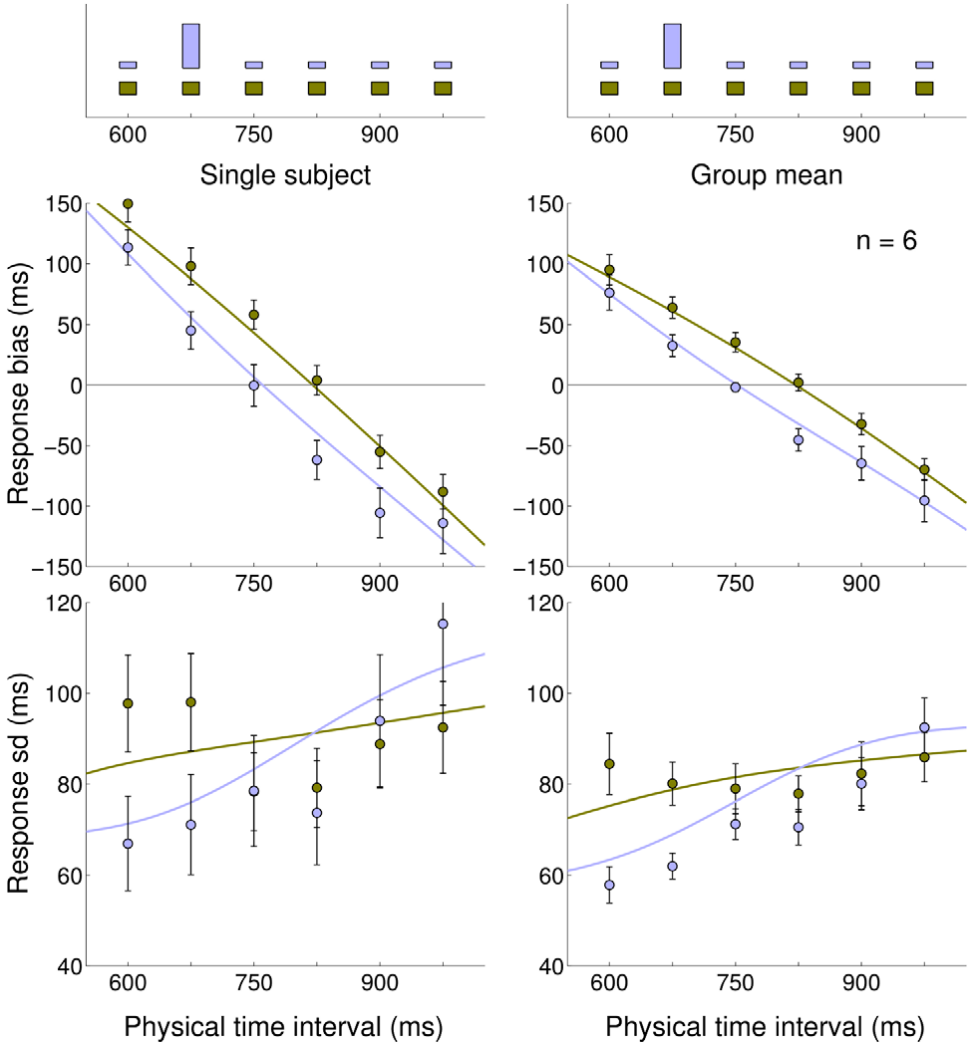
Experiment 2: Medium Uniform and Medium Peaked blocks. *Very top:* Experimental distributions for Medium Uniform (light brown) and Medium Peaked (light blue) blocks, repeated on top of both columns. *Left column:* Mean response bias (average difference between the response and true interval duration, top) and standard deviation of the response (bottom) for a representative subject in both blocks (light blue: Medium Uniform; light brown: Medium Peaked). Error bars denote s.e.m. Continuous lines represent the Bayesian model ‘fit’ obtained averaging the predictions of the most supported models (Bayesian model averaging). *Right column:* Mean response bias (top) and standard deviation of the response (bottom) across subjects in both blocks (mean 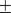 s.e.m. across subjects). Continuous lines represent the Bayesian model ‘fit’ obtained averaging the predictions of the most supported models across subjects.

**Figure 5:**
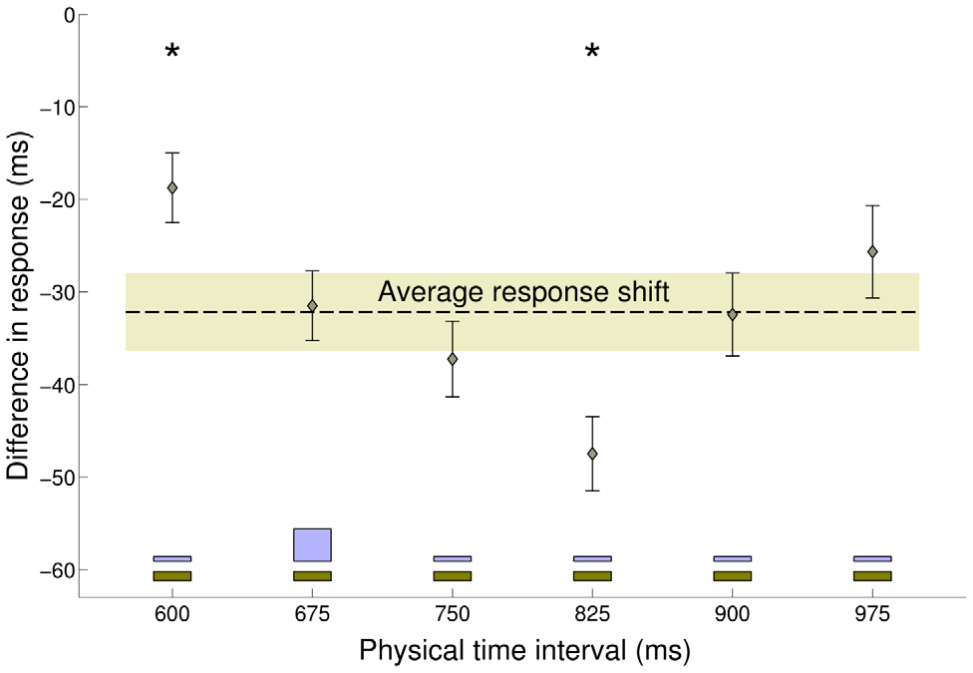
Experiment 2: Difference in response between Medium Peaked and Medium Uniform blocks. Difference in response between the Medium Peaked and the Medium Uniform conditions as a function of the actual interval, averaged across subjects (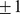 s.e.m.). The experimental distributions (light brown: Medium Uniform; light blue: Medium Peaked) are plotted for reference at bottom of the figure. The dashed black line represents the average response shift (difference in response between blocks, averaged across all subjects and stimuli), with the shaded area denoting 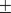 s.e.m. The average response shift is significantly different from zero (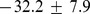 ms; two-sample t-test 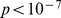), meaning that the two conditions elicited consistently different performance. Additionally, the responses were subject to a ‘local’ (i.e. interval-dependent) modulation superimposed to the average shift, that is, intervals close to the peak of the distribution (675 ms) were attracted towards it, in addition to the average shift, while intervals far away from the peak were less affected. (*) The response shift at 600 ms and 825 ms is significantly different from the average response shift; 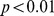.

Examination of the responses showed a central tendency as encountered in the previous experiment (Figure 4 top). However, despite the identical range of intervals in both blocks, subjects were sensitive to the relative probability of the intervals [27]. In particular, the responses in the Peaked block (light blue points) appeared to be generally shifted towards shorter durations and this shift was interval dependent (see Figure 5). This behavior is qualitatively consistent with a simple Bayesian inference process, according to which the responses are ‘attracted’ towards the regions of the prior distribution with greatest probability mass. Intuitively, the average (‘global’) shift of responses can be thought of as arising from the shift in the distribution mean, from the Uniform distribution (mean 787.5 ms) to the Peaked distribution (mean 731.3 ms); whereas interval-dependent (‘local’) effects are a superimposed modulation by the probability mass assignments of the distribution. This is only a simplified picture, as the biases depend on a nonlinear inference process, which is also influenced by other details of the Bayesian model (such as the loss function), but the qualitative outcome is likely to be similar in many relevant cases.

The standard deviation of the responses showed a significant decrease in variability around the peak for the Peaked condition (Figure 4 bottom; two-sample F-test 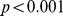). This effect could be simply due to practice as subjects received feedback more often at peak intervals, however the local modulation of bias previously described (Figure 5) suggests a Bayesian interpretation. In fact, because of the local ‘attraction’ effect, interval durations close to the peak would elicit responses that map even closer to it, therefore compressing the perceptual variability, an example of bias-variance trade-off [6].

The results of the second experiment show that people take into account the different nature of the two experimental distributions, in agreement with previous work that found differential effects in temporal reproduction for skewed vs uniform distributions of temporal intervals on a wider, suprasecond range [27]. The performance of the subjects in the two blocks is consistent with a Bayesian ‘attraction’ in the response towards the intervals with higher prior probability mass. Moreover, although the average negative shift in the response observed in the Peaked condition versus the Uniform one might be compatible with a temporal recalibration effect that shortens the perceived duration between action and effect [11], [28], [29], the interval-dependent bias modulation (Figure 5) and the reduction in variability around the peak (Figure 4 bottom) suggest there may instead be in this case a Bayesian explanation.

In order to address more specific, quantitative questions about our results we set up a formal framework based on a Bayesian observer and actor model.

### Bayesian observer model

We modelled the subjects' performance with a family of Bayesian ideal observer (and actor) models which incorporated both the perception (time interval estimation) and action (reproduction) components of the task; see Figure 2 (bottom) for a depiction of the generative model of the data. We assume that on a given trial a time interval 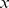 is drawn from a probability distribution 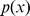 (the experimental distribution) and the observer makes an internal measurement 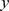 that is corrupted by sensory noise according to the *sensory likelihood* 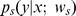, where 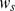 is a parameter that determines the sensory (estimation) variability. Subjects then reproduce the interval with a motor command of duration 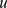. This command is corrupted by motor noise, producing the response duration 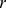 – the observed reproduction time interval – with conditional probability density 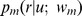 (the *motor likelihood*), with 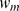 a motor (reproduction) variability parameter. Subjects receive an error specified by a mapping 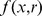 and we assume they try to minimize a (quadratic) loss based on this error.

In our model we assume that subjects develop an internal estimate of both the experimental distribution and error mapping (the feedback associated with a response 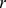 to stimulus 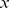), which leads to the construction of a (subjective) prior, 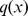, and subjective error mapping 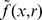; the latter is then squared to obtain the loss function. This allows the prior and subjective error mapping to deviate from their objective counterparts, respectively 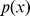 and 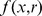.

Following Bayesian decision theory, the ‘optimal’ action 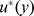 is calculated as the action 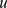 that minimizes the subjectively expected loss:
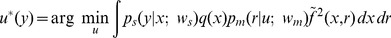
(1)where the integral on the right hand side is proportional to the subjectively expected loss. Combining Eq. 1 with the generative model of Figure 2 (bottom) we computed the distribution of responses of an ideal observer for a target time interval 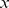, integrating over the hidden internal measurement 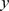 which was not directly accessible in our experiment.

Therefore the reproduction time 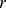 of an ideal observer, given the target interval 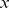, is distributed according to:
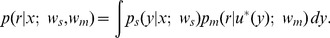
(2)
Eqs. 1 and 2 are the key equations that allow us to simulate our task, in particular by computing the mean response bias and standard deviation of the response for each interval (Section 1 in Text S1). Eq. 1 represents the internal model and deterministic decision process adopted by the subject whereas Eq. 2 represents probabilistically the objective generative process of the data. Notice that the experimental distribution 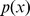 and objective error mapping 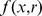 do not appear in any equation: the distribution of responses of ideal observers only depends on their internal representations of prior and loss function.

**Figure 6:**
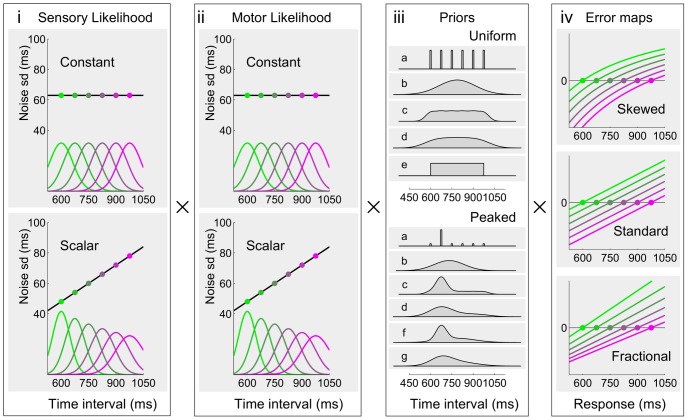
Bayesian observer and actor model components. Candidate (**i**) sensory and (**ii**) motor likelihoods, independently chosen for the sensory and motor noise components of the model. The likelihoods are Gaussians with either constant or ‘scalar’ (i.e. homogeneous linear) variability. The amount of variability for the sensory (resp. motor) component is scaled by parameter 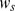 (resp. 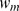). **iii**) Candidate priors for the Medium Uniform (top) and Medium Peaked (bottom) blocks. The candidate priors for the Short Uniform (resp. Long Uniform) blocks are identical to those of the Medium Uniform block, shifted by 150 ms in the negative (resp. positive) direction. See Methods for a description of the priors. **iv**) Candidate subjective error maps. The graphs show the error as a function of the response duration, for different discrete stimuli (drawn in different colors). From top to bottom: Skewed error 
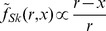
; Standard error 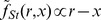; and Fractional error 
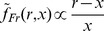
. The scale is irrelevant, as the model is invariant to rescaling of the error map. The squared subjective error map defines the loss function (as per Eq. 1).

Eqs. 1 and 2 describe a *family* of Bayesian observer models, a single Bayesian ideal observer is fully specified by picking (i) a noise model for the sensory estimation process, 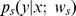; (ii) a noise model for the motor reproduction process 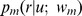; (iii) the form of the prior 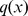; and (iv) the loss function 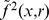 (Figure 6 and Methods). To limit model complexity, in the majority of our analyses we used the same likelihood functions (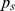, 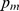 and their parameters 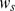, 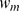) for both the generative model (Eq. 2) and the internal model (Eq. 1). Analogously, for computational reasons in our basic model we assumed a quadratic exponent for the loss function (Eq. 1); in a subsequent analysis we relaxed this requirement (Section 2 in Text S1).

### Bayesian model comparison

To study the nature of the internal model adopted by the participants, we performed a full Bayesian model comparison over the family of Bayesian ideal observer models. For each participant we assumed that the sensory and motor noise, the approximation strategy for the priors, and the loss function were shared across different experimental blocks. The model comparison was performed over a discrete set of model components, that is, possible choices for the priors, loss functions and shape of likelihoods (Figure 6). In particular, priors and loss functions did not have continuous parameters, as a parametric model would likely be ambiguous or hard to interpret, with multimodal posterior distributions over the parameters (as multiple combinations of likelihoods, prior and cost function can make identical predictions). Instead, we considered a finite number of parameter-free models of loss function, prior and shape of likelihoods, leaving only two continuous parameters for characterizing the sensory and motor variability.

Both sensory and motor noise were modelled with Gaussian distributions whose means were centered on the interval and whose standard deviations could either be constant or ‘scalar’, that is, grow linearly with the interval (Figure 6 i and ii). We used two parameters, 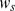 and 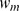, which represent the coefficient of variation of the subject's sensory and motor noise. For the scalar case this simply specifies the coefficient of proportionality of the standard deviation with respect to the mean, whereas in the constant case it specifies the proportion of noise with respect to a fixed interval (787.5 ms).

We considered three different possible subjective error metrics corresponding to the Skewed error 
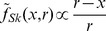
 (the error map we provided experimentally), the Standard error 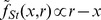, and a Fractional error 
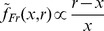
 (Figure 6 iv), which were then squared to obtain the loss function (see also Methods). Note that scaling these mappings does not change the optimal actions and hence the model selection process.

We compared different approximation schemes for the priors, such as the true discrete distribution (Figure 6 iii, a) or a single Gaussian whose mean and standard deviation matched those of the true prior (b). We also considered two smoothed versions of the experimental distribution with a weak (c) and strong (d) smoothing parameter, or some other block-dependent approximations, e.g. for the Uniform blocks we considered a uniform distribution over the stimulus range (e); see Methods for a full description. To constrain the model selection process, we assumed that subjects adopted a consistent approximation scheme across blocks.

For each participant we computed the support for each model based on the psychophysical data, that is the posterior probability of the model, Pr(model| data). Assuming an a priori indifference among the models, this corresponds (up to a normalization factor) to the model marginal likelihood Pr(data| model), which was obtained by numerical integration over the two-dimensional parameter space (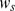 and 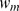).

We then calculated the Bayesian model average for the response mean bias and standard deviation, shown by the continuous lines in Figure 3 and 4. Note that the Bayesian model ‘fits’ are obtained by computing the marginal likelihood of the models and integrating the model predictions over the posterior of the parameters (model averaging), with no parameter fitting. The mean biases fits show a good quantitative match with the group averages (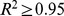 for all blocks); the standard deviations are typically more erratic and we found mainly a qualitative agreement, as observed in previous work [6].

For each participant of Experiments 1 and 2 we computed the most probable (i) sensory and (ii) motor likelihoods, (iii) priors and (iv) loss function (Table S1). The model comparison confirmed that the best noise models were represented by the ‘scalar’ variability, which had relevant support for both the sensory component (7 subjects out of 10) and the motor component (8 subjects out of 10). This result is consistent with previous work in both the sensory and motor domain [5], [6], [25], [30]. The most supported subjective error map was the Skewed error (7 subjects out of 10), which matched the feedback we provided experimentally. The priors most supported by the data were typically smooth, peaked versions of the experimental distributions. In particular, according to the model comparison, almost all subjects (9 out of 10) approximated the discrete uniform distributions in the Uniform blocks with normal distributions (same mean and variance as the true distribution; Figure 6 iii top, b). However, in Experiment 2 most people (5 out of 6) seemed to approximate the experimental distribution in the Peaked block not with a standard Gaussian, but with a skewed variant of a normal distribution (Figure 6 iii bottom, d, f and g), suggesting that their responses were influenced by higher order moments of the true distribution and not just the mean and variance (see Discussion).

For Experiment 2 we also relaxed some constraints on the priors, allowing the model selection to pick a Medium Uniform prior for the Medium Peaked block and vice versa. Nevertheless, the model comparison showed that the most supported models were still the ones in which the priors matched the block distribution, supporting our previous findings that subjects' responses were consistent with the temporal context and changed when switching from one block to another (as visible in Figure 4).

### Nonparametric reconstruction of the priors

**Figure 7:**
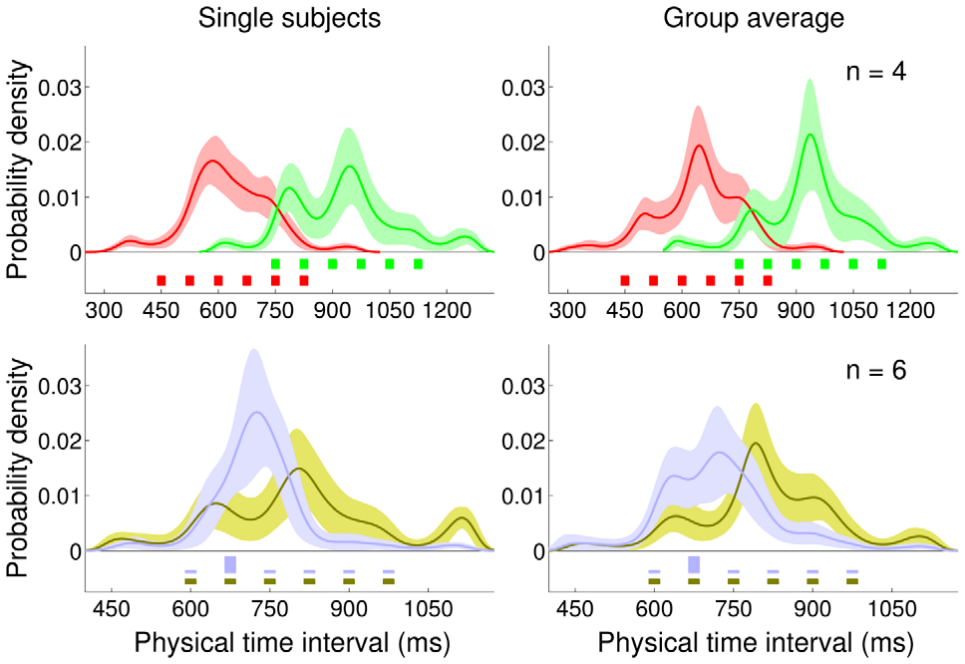
Nonparametrically inferred priors (Experiment 1 and 2). *Top row:* Short Uniform (red) and Long Uniform (green) blocks. *Bottom row:* Medium Uniform (light brown) and Medium Peaked (light blue) blocks. *Left column:* Nonparametrically inferred priors for representative participants. *Right column:* Average inferred priors. Shaded regions are 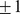 s.d. For comparison, the discrete experimental distributions are plotted under the inferred priors.

To study in detail the internal representations, we relaxed the constraint on the priors. Rather than choosing from a fixed set of candidate priors (Figure 6 iii), we allowed the prior to vary over a much wider class of smooth, continuous distributions. We assumed that the noise models and loss function emerging from the model comparison were a good description of the subjects' decision making and sensorimotor processing in the task. We therefore fixed these components of the observer's model and inferred nonparametrically, on an individual basis, the shape of the priors most compatible with the measured responses (Figure 7; see Methods for details).

Examination of the recovered priors shows that the subjective distributions were significantly different from zero only over the range corresponding to the experimental distribution, with only occasional tails stretching outside the interval range (e.g. Figure 7 bottom left). This suggests that in general people were able to localize the stimulus range in the blocks. The priors did not typically take a bell-like shape, but rather we observed a more or less pronounced peak at the mean of the true distribution, with the remaining probability mass spread over the rest of the range. Interestingly, the group averages for the Uniform priors over the Short, Medium and Long ranges (Figure 7 top right, both, and bottom right, light brown) exhibit very similar, roughly symmetrical shapes, shifted over the appropriate stimulus range. Conversely, the Peaked prior (Figure 7 bottom right, light blue) had a distinct, skewed shape.

To compare the inferred priors with the true distribution, we calculated their distribution moments (Table 2). We found that the first three moments of the inferred priors (in the table reported as mean, standard deviation and skewness) were statistically indistinguishable from those of the true distributions for all experimental conditions (Hotelling's multivariate one-sample 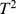 test considering the joint distribution of mean, standard deviation and skewness against the true values; 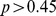 for all blocks). This result confirmed the previously stated hypothesis that participants had developed an internal representation which included higher order moments and not just the mean and variance of the experimental distribution. However, when including the fourth moment (kurtosis) in the analysis, we observed a statistically significant deviation of the recovered priors with respect to the true distributions (Hotelling's 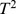 test with the joint distribution of the first four moments; 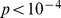 for all blocks); in particular, the inferred priors seem to have more pronounced peaks and/or heavier tails. First of all, note that the heightened kurtosis is not an artifact due to the averaging process across subjects or the sampling process within subjects, as we averaged the moments computed for each sampled distribution (see Methods) rather than computing the moments of the average distribution. In other words, all recovered priors are (on average) heavy tailed, it's not just the mean prior that it is ‘accidentally’ heavy tailed as a mixture of light-tailed distributions. So this result could mean that the subjects' internal representations are actually heavy-tailed, for instance to allow for unexpected stimuli. However, there could be a simpler explanation that the presence of outliers arise from occasional trivial mistakes of the participants. We, therefore, considered a straightforward extension of our model which added the possibility of occasional ‘lapses’ with a lapse rate 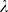, where the response in a lapse trial is simply modelled as a uniform distribution over a wide range of intervals (Section 3 in Text S1). In terms of marginal likelihood, generally the models with lapse performed better than the original models, but with no qualitative difference in the preferred model components. Crucially, we did not observe a significant change in the kurtosis of the recovered priors, ruling out the possibility that the heightened kurtosis had been caused by trivial outliers.

**Table 2:** Main statistics of the experimental distributions and nonparametrically inferred priors (Experiment 1 and 2; Skewed feedback).

	Short Uniform				Long Uniform			
	Objective	Subjective			Objective	Subjective		
Mean (ms)	637.5	644.2	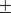	12.8	937.5	929.9	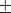	19.6
Std (ms)	128.1	117.4	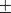	13.3	128.1	131.2	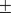	16.9
Skewness	0	−0.17	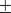	0.24	0	−0.12	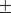	0.41
Ex. Kurtosis	−1.27	0.86	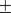	1.24	−1.27	0.82	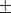	0.98

Comparison between the main statistics of the ‘objective’ experimental distributions and the ‘subjective’ priors nonparametrically inferred from the data. The subjective moments are computed by averaging the moments of sampled priors pooled from all subjects (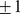 s.d.); see Figure 7, right column and Methods for details. In statistics, the excess kurtosis is defined as kurtosis 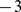, such that the excess kurtosis of a normal distribution is zero. Heavy tailed distributions have a positive excess kurtosis.

Our analysis therefore showed that, according to the inferred priors, people generally acquired internal representations that were smooth, heavy-tailed approximations to the experimental distributions of intervals, in agreement up to the first three moments.

### Experiment 3: Effect of the shape of feedback on the loss function

In our ideal observer model we compared three candidate loss functions: Skewed, Standard and Fractional (Figure 6 iv). The results of the model comparison in the first two experiments with Skewed feedback showed that there was a good match between experimentally provided feedback and subjective error metric. However, we could not rule out the possibility, albeit unlikely, that participants were ignoring the experimental feedback and following an internal error signal that just happened to be similar in shape to the Skewed error. We therefore performed an additional experiment to verify that subjects behavior is driven by the feedback provided.

**Figure 8:**
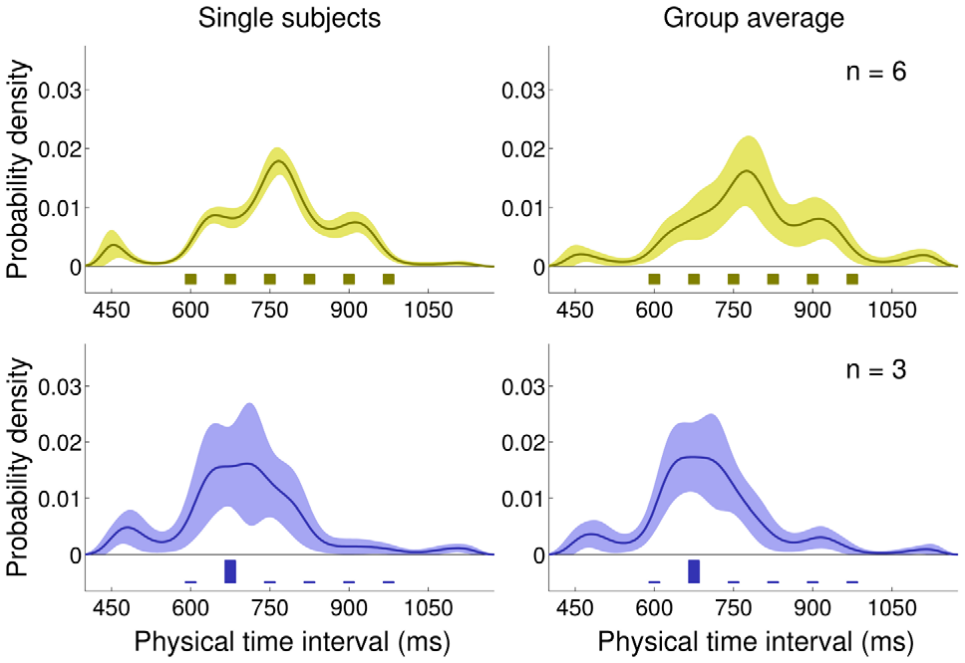
Nonparametrically inferred priors (Experiment 3 and 4). *Top row:* Medium Uniform (light brown) block. *Bottom row:* Medium High-Peaked (dark blue) block. *Left column:* Nonparametrically inferred priors for representative participants. *Right column:* Average inferred priors. Shaded regions are 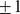 s.d. For comparison, the discrete experimental distributions are plotted under the inferred priors.

We again used a Medium Uniform block but now with Standard error 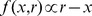 as feedback (see Figure S5 in Text S2). The model comparison for this group showed that the responses of 4 subjects out of 6 were best explained with a Standard loss function. Moreover, no subject appeared to be using the Skewed loss function (Table S1). These results confirm that most people correctly integrate knowledge of results with sensory information in order to minimize the average (squared) error, or an empirically similar metric. Furthermore, all inferred individual priors showed a remarkable agreement with a smoothed approximation of the experimental distribution of intervals (Figure 8 top), suggesting that the Standard error feedback may be easier to use for learning. As in the previous experiments, the average moments of the inferred priors (up to skewness) were statistically indistinguishable from those of the true distribution, with a significant difference in the kurtosis (Table 3 left; Hotelling's 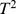 test, first three moments: 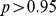; first four moments: 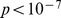).

**Table 3:** Main statistics of the experimental distributions and nonparametrically inferred priors (Experiment 3 and 4; Standard feedback).

	Medium Uniform				Medium High-Peaked			
	Objective	Subjective			Objective	Subjective		
Mean (ms)	787.5	782.6	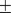	18.7	703.1	702.0	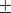	17.9
Std (ms)	128.1	131.7	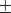	13.6	80.5	119.5	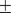	17.9
Skewness	0	0.03	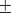	0.30	2.25	0.67	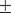	0.37
Ex. Kurtosis	−1.27	0.42	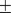	0.53	−0.86	1.66	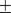	1.32

Comparison between the main statistics of the ‘objective’ experimental distributions and the ‘subjective’ priors nonparametrically inferred from the data. The subjective moments are computed by averaging the moments of sampled priors pooled from all subjects (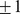 s.d.); see Figure 8, right column and Methods for details.

### Experiment 4: High-Peaked distribution

In the Peaked block we did not observe any significant divergence from the Bayesian prediction. However, the ratio of presentations of ‘peak’ intervals (675 ms) to the others was low (1.4) and possibly not enough to induce other forms of temporal adaptation [29], [31]. To examine whether we might see deviations from Bayesian integration for larger ratios we therefore tested another group of subjects on a more extreme variant of the Peaked distribution in which the peak stimulus had a probability of 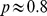 and therefore a ratio of about 4.0. We provided feedback through the Standard error mapping, as the previous experiment had showed that subjects can follow it at least as well as the Skewed mapping.

Due to the large peak interval presentation frequency we had fewer test data points in the model fitting. Therefore, we constrained the model comparison by only considering the Standard loss in order to prevent the emergence of spurious model components capturing random patterns in the data. We found that the recovered internal priors were in good qualitative agreement with the true distribution, with statistically indistinguishable means (Figure 8 bottom, and Table 3; one sample two-tailed t-test 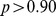). When variance and higher moments were included in the analysis, though, the distributions were significantly different (Hotelling's 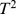 test, mean and variance: 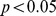; first three moments: 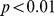; first four moments: 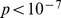) suggesting that the distribution may have been ‘too peaked’ to be learnt exactly; see Discussion. Nevertheless, the observed biases of the responses were well explained by the basic Bayesian models (group mean: 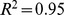), and the standard deviations were in qualitative agreement with the data (Figure S6 in Text S2).

### Experiment 5: Bimodal distributions

Our previous experiments show that people are able to learn good approximation of flat or unimodal distributions of intervals relatively quickly (a few sessions), under the guidance of corrective feedback. Previous work in sensorimotor learning [15] and motion perception [32] has shown that people can learn bimodal distributions. Whether the same is attainable for temporal distributions is unclear; a recent study of time interval reproduction [27] obtained less definite results with a bimodal ‘V-shaped’ distribution, although training might have been too short, as subjects were exposed only to 120 trials in total and without performance feedback.

To examine whether subjects could easily learn bimodality of a temporal distribution with the help of feedback we tested two new groups of subjects on bimodal distributions of intervals on a Medium range (600–975 ms, as before) and on a Wide range (450–1125 ms), providing in both cases Standard feedback. In the Medium Bimodal block the intervals at 600 and 975 ms had each probability 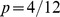, whereas the other four middle intervals (675, 750, 825, 900 ms) had each probability 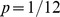. In the Wide Bimodal block the six ‘extremal’ intervals (450, 525, 600 ms and 975, 1050, 1125 ms) had each probability 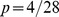 whereas the middle intervals had probability 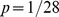. Note that in both cases extremal intervals were four times as frequent as middle intervals.

**Figure 9:**
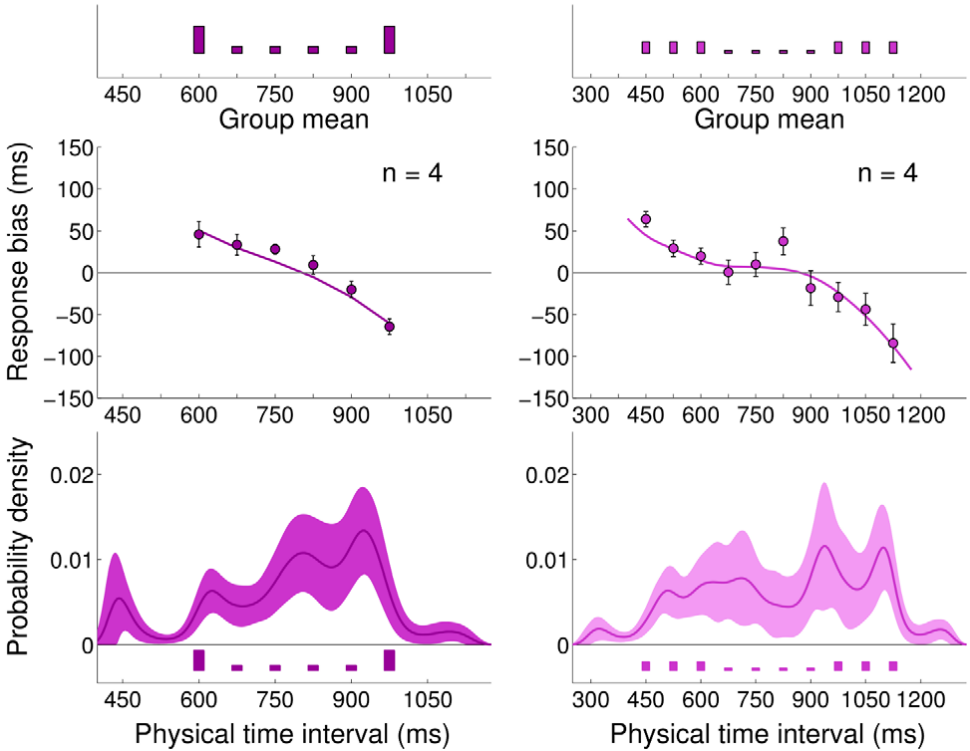
Experiment 5: Medium Bimodal and Wide Bimodal blocks, mean bias and nonparametrically inferred priors. *Very top:* Experimental distributions for Medium Bimodal (dark purple, left) and Wide Bimodal (light purple, right) blocks. *Top:* Mean response bias across subjects (mean 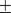 s.e.m. across subjects) for the Medium Bimodal (left) and Wide Bimodal (right) blocks. Continuous lines represent the Bayesian model ‘fit’ obtained averaging the predictions of the most supported models across subjects. *Bottom:* Average inferred priors for the Medium Bimodal (left) and Wide Bimodal (right) blocks. Shaded regions are 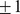 s.d. For comparison, the experimental distributions are plotted again under the inferred priors.

In the Medium Bimodal block, subjects' responses exhibited a typical central tendency effect (Figure 9 top left) which suggests that people did not match the bimodality of the underlying distribution. To constrain the model comparison we inferred the subjects' priors under the assumption of scalar sensory and motor noise models and Standard loss function, as found by our previous analyses. As before, we first used a discrete set of priors (see Methods) that we used to compute the model ‘fit’ to the data and then we performed a nonparametric inference. The nonparametrically inferred priors for the Medium Bimodal distribution (Figure 9 top right) suggest that on average subjects developed an internal representation that differed from those seen in previous experiments and, as before, we found a good agreement between moments of the experimental distribution and moments of the inferred priors up to skewness (Table 4 left). However, results of the Bayesian model comparison among a discrete class of flat, unimodal or bimodal priors do not support the hypothesis that subjects actually learnt the bimodality of the experimental distribution (data not shown). Part of the problem may have been that in the Medium Bimodal distribution the two modes were relatively close, and due to sensory and motor uncertainty subjects could not gather enough evidence that the experimental distribution was not unimodal (but see Discussion). We repeated the experiment therefore on a wider range with a different group of subjects.

**Table 4:** Main statistics of the experimental distributions and nonparametrically inferred priors for bimodal distributions (Experiment 5; Standard feedback).

	Medium Bimodal				Wide Bimodal			
	Objective	Subjective			Objective	Subjective		
Mean (ms)	787.5	794.5	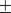	34.2	787.5	822.1	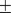	70.7
Std (ms)	160.6	155.7	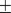	37.2	251.6	219.2	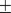	29.3
Skewness	0	−0.33	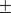	0.39	0	−0.22	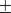	0.57
Ex. Kurtosis	−1.72	−0.08	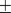	0.90	−1.64	−0.40	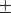	0.51

Comparison between the main statistics of the ‘objective’ experimental distributions and the ‘subjective’ priors nonparametrically inferred from the data. The subjective moments are computed by averaging the moments of sampled priors pooled from all subjects (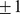 s.d.); see Figure 9, bottom and Methods for details.

The pattern of subjects' responses in the Wide Bimodal block shows a characteristic ‘S-shaped’ bias profile (Figure 9 top right) which is compatible with either a flat or a slightly bimodal prior. The nonparametrically inferred priors for the Wide Bimodal distribution (Figure 9 bottom right) again suggest that on average subjects acquired, albeit possibly with less accuracy (Table 4 right), some broad features of the experimental distribution; however individual datasets are quite noisy and again we did not find strong evidence for learning of bimodality.

Our results with bimodal distributions confirm our previous finding that people seem to be able to learn broad features of experimental distributions of intervals (mean, variance, skewness) with relative ease (a few sessions of training with feedback). However, more complex features (kurtosis, bimodality) seem to be much harder to learn (see Discussion).

## Discussion

Our main finding is that humans, with the help of corrective feedback, are able to learn various statistical features of both simple (uniform, symmetric) and complex (peaked, asymmetric or bimodal) distributions of time intervals. In our experiments, the inferred internal representations were smooth, heavy tailed approximations of the experimental distributions, in agreement typically up to third-order moments. Moreover, our results suggest that people take into account the shape of the provided feedback and integrate it with knowledge of the statistics of the task in order to perform their actions.

The statistics of the responses of our subjects in the Uniform blocks were consistent with results from previous work; in particular, we found biases towards the mean of the range of intervals (central tendency) [6], [8], [26], [33] and the variability of the responses grew roughly linearly in the sample interval duration (scalar property) [6], [34]. The responses in the Peaked and High-Peaked blocks showed analogous biases, but they were directed towards the mean of the distribution rather than the mean of the range of intervals (the two means overlapped in the Uniform case) [27]. We also observed a significant reduction in variability at the peak. These results were sufficient to suggest that subjects considered the temporal statistics of the context in their decision making processes. We found a similar regression to the mean for a ‘narrow’ bimodal distribution (Medium Bimodal), in qualitative agreement with previous work that found a simple central tendency with a ‘V-shaped’ temporal distribution [27] (although with very limited training, no feedback and a suprasecond range). However, for a bimodal distribution on a wider range we observed ‘S-shaped’ biases which seem compatible with a nonlinear decision making process [15]. However, more refined conclusions needed the support of a formal framework.

### Bayesian model

Our modelling approach consisted of building a family of Bayesian observer and actor models, which provided us with a mathematical structure in which to ask specific questions about our subjects [35], going beyond mere statements about Bayesian optimality. In particular, we were interested in (1) whether people would be able to learn nontrivial temporal distributions of intervals and what approximations they might use, and (2) how their responses would be affected by performance feedback. Our observer model resembled the Bayesian Least Squares (BLS) observer described in [6], but it explicitly included an action component as part of the internal model. Moreover, to answer (1) we allowed the prior to differ from the experimental distribution, and to study (2) we considered additional shapes for the loss function in addition to the Standard squared loss 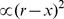.

The Bayesian model comparison gave us specific answers for each of our subjects, and a first validation came from the success of the most supported Bayesian observer and actor models in capturing the statistics of the subjects' responses in the task. However, goodness of fit per se is not necessarily an indicator that the components found by the model comparison reflected true findings about the subjects, rather than ‘overfitting’ arbitrary statistical relationships in the data. This is of particular relevance for Bayesian models, because of the underlying degeneracy among model components [21].

Our approach consisted in considering a large, ‘reasonable’ set of observer models that we could link to objective features of the experiment. This does not solve the degeneracy problem per se but it prevents the model comparison from finding arbitrary solutions. In particular, the set of experiments was designed in order to provide evidence that each element of the model mapped on to an experimentally verifiable counterpart; crucially, we found that a change in a component of the experimental setup (e.g. experimental distribution and feedback) correctly induced a switch in the corresponding inferred component of the model (prior and loss function). We also avoided overfitting by limiting our basic models to only two continuous noise parameters, which were then computed through model averaging and further validated by independent direct measures.

To further validate our methods, we directly measured the subject's noise parameters (sensory and motor noise, 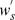 and 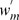) in separate tasks and compared them with the model parameters 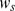, 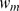 inferred from the main experiments (see Section 4.1 in Text S1 for full description). The rationale is that, in an idealized situation, we would be able to measure some features of the subjects with an objective, independent procedure and the same features would be predictive of the individual performances in related tasks [16]. The measured parameters were highly predictive of the group behavior, and reasonably predictive at the individual level for the sensory parameter, confirming that the model parameters were overall correctly representing objective ‘noise properties’ of the subjects.

Overall, our modelling techniques were therefore validated by (a) goodness of fit, (b) consistency between inferred model components and experimental manipulations, and (c) consistency between the model parameters and independent measurements of the same quantities.

### Comparison between inferred priors and experimental distributions

Given the validation of the results of the model comparison, we performed a nonparametric inference of the priors acquired by participants during the task. Other recent works have inferred the shape of subjective ‘natural’ perceptual priors nonparametrically, such as in visual orientation [24] and speed [36] perception, but studies that focussed on experimentally acquired priors mostly recovered them under parametric models (e.g. Gaussian priors with variable mean and variance) [35], [37]–[39]. The nonparametric method allowed us to study the accuracy of the subjects in learning the experimental distributions, comparing summary statistics such as the moments of the distributions up to fourth order. Note that the significance and reliability of the recovered priors is based on the correctness of our assumptions regarding the observer and actor model; unconstrained priors might capture all sorts of statistical details, one of the typical objections to Bayesian modelling [40]. However, by dividing the model selection stage (and its validation) from the prior reconstruction process we prevented the most pathological forms of overfitting.

The internal representations inferred from the data show a good agreement with the central moments of the true distributions typically up to third order (mean, variance and skewness). Subjects however showed some difficulties in learning variance and skewness when the provided distribution was extremely peaked, with a width less than the subjects' perceptual variability. This discrepancy observed in the High-Peaked block may have arisen because (a) the experimental distribution's standard deviation was equal or lower in magnitude compared to the perceptual variability of the subjects (experimental distribution standard deviation: 80.5 ms; subject's average sensory standard deviation at the mean of the distribution: 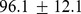 ms; mean 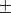 sd across subjects) and (b) due to the shape of the distribution, subjects had much less practice with intervals away from the peak. Another explanation is that subjects' representation of relative frequencies of different time intervals was systematically distorted, with overestimation of small relative frequencies and underestimation of large relative frequencies (see [41] for a critical review), but note that this would arguably produce a change in the mean of the distribution as well, which we did not observe.

Moreover, the recovered priors in all blocks had systematically heavier tails (higher kurtosis) than the true distributions. By exploring an extended model that included lapses we ruled out that this particular result was due to trivial outliers in our datasets. However, our results are compatible with other more sophisticated reasons for the heavy tails we recovered, in particular (a) the objective likelihoods might be non-Gaussian, with heavier tails [42], and (b) the loss functions might follow a less-than-quadratic power law [43], hypothesis for which we found some evidence, although inconclusive, by studying observer models with non-quadratic loss functions (Section 2 in Text S1). Experimentally, both (a) and (b) would imply that in our datasets there would be more outliers than we would expect from a Gaussian noise model with quadratic losses.

Our experiments with bimodal distributions show that, although people's responses were affected by the experimental distribution of intervals in a way which is clearly different from our previous experiments with uniform or peaked distributions, the inferred priors in general fail to capture bimodality and are consistent instead with a broad uniform or multimodal prior (where the peaks however do not necessarily fall at the right places). Note that the average sensory standard deviation for subjects in Experiment 5 was 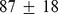 ms (Medium Bimodal; mean 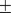 sd across subjects) and 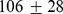 ms (Wide Bimodal), calculated at the center of the interval range. In other words, in both blocks, the centers of the peaks were well-separated in terms of perceptual discriminability (on average by at least four standard deviations). This suggests that most subjects did not simply fail to learn the bimodality of the distributions because they had problems distinguishing between the two peaks.

### Temporal recalibration and feedback

Lag adaptation is a robust phenomenon for which the perceived duration between two inter-sensory or motor-sensory events shortens after repeated exposure to a fixed lag between the two [10], [11], [44]; see [45] for a review. It is currently uncertain whether lag adaptation is a ‘global’ temporal recalibration effect (affecting all intervals) [46], ‘local’ (affecting only intervals in a neighborhood of the adapter lag) [47], or both. What is clear is that lag adaptation cannot be interpreted as a Bayesian effect in terms of prior expectations represented by the sample distribution of adaptation and test intervals, since its signature is a ‘repulsion’ from the adapter as opposed to the ‘attraction’ induced by a prior [4], [47], [48].

Our experimental setup for the peaked blocks mimicked the distributions of intervals of typical lag adaptation experiments [11], [29], with the adapter interval set at 675 ms (the ‘peak’). However, we did not detect any noticeable disagreement with the predictions of our Bayesian observer model and, in particular, there was no significant ‘repulsion effect’ from the peak, neither global nor local. Our results suggest that people are not subject to the effects of lag adaptation, or can easily compensate for them, in the presence of corrective feedback.

Sensorimotor lag adaptation seems to belong to a more general class of phenomena of temporal recalibration which induce an adjustment of the produced (or estimated) timing of motor commands to meet the goals of the task at hand. In the case of experimentally induced actuator delays in a time-critical task, such as controlling a spaceship through a minefield in a videogame [49] or driving a car in a simulated environment [50], visual temporal information about delays provides an obvious, compelling reason to recalibrate the timing of actions. However, feedback regarding timing performance need not be provided only in temporal ways. Previous studies have shown that people take into account performance feedback (knowledge of results) when the feedback about the timing of their motor response is provided in various ways, such as verbal or visual report in milliseconds [23], [51] or bars of variable length [52]. Interestingly, people tend to also follow ‘erroneous’ feedback [52]–[54]. However, this can be explained by the fact that people's behavior in a timing task is goal-oriented (e.g. minimizing feedback error), and therefore these experiments suggest that people are able to follow external, rather than erroneous, feedback. In fact, when participants are told that feedback might sometimes be incorrect, which corresponds to setting different expectations regarding the goal of the task, they adjust their timing estimates taking feedback less into account [53]. Ambiguity regarding the goal of a timing task with non-obvious consequences – as opposed to actions that have obvious sensorimotor consequences, such as catching a ball – can be reduced by imposing an explicit gain/loss function [5], [55], and it has been found that people can act according to an externally presented asymmetric cost (even though their timing behavior is not necessarily ‘optimal’ [55]).

Our work extends these previous findings by performing a model comparison with different types of symmetric and asymmetric loss functions and providing additional evidence that most people are able to correctly integrate an arbitrary external feedback in their decision process, while executing a sensorimotor timing task, so to minimize the feedback error.

### Bayesian sensorimotor timing

There is growing evidence that many aspects of human sensorimotor timing can be understood in terms of Bayesian decision theory [3], [5], [6]. The mechanism through which people build time estimates, e.g. an ‘internal clock’, is still unclear (see [56] for a review), but it has been proposed that observers may integrate both internal and external stochastic sources of temporal information in order to estimate the passage of time [7], [57].

Inspired by these results, in our work we assumed that people build an internal representation of the temporal distribution of intervals presented in the experiment. However, for all timing tasks in which more or less explicit knowledge of results is given to the subjects (e.g. ours, [6], [26]), an alternative explanation is that people simply learn a mapping from a duration measurement to a given reproduction time (strategy known as *table look-up*), with no need of learning of a probability distribution [58]. At the moment we cannot completely discard this possibility, but other timing studies have shown that people perform according to Bayesian integration even in the absence of feedback both for simple [4], [8] and possibly skewed distributions [27], suggesting that people indeed take into account the temporal statistics of the task in a context-dependent way. Moreover, previous work in motor learning in the spatial domain has shown that people do not simply learn a mapping from a stimulus to a response, but adjust their performance according to the reliability of the sensory information [15], a signature of probabilistic inference [59]. Analogous findings have been obtained in multisensory integration [18], [60], [61] and for visual judgements (‘offset’ discrimination task) under different externally imposed loss functions [20], crucially in all cases without knowledge of results. All these findings together support the idea that sensorimotor learning follows Bayesian integration, also in the temporal domain. However, the full extent of probabilistic inference in sensorimotor timing needs further study, possibly involving transfer between different conditions in the absence of knowledge of results [58].

Our results answer some of the questions raised in [6], in particular about the general shape of the distributions internalized by the subjects and the influence of feedback on the responses. An avenue for further work is related to the detailed profile of the likelihoods and possible departures from the scalar property [34], [62] (see also Section 4 in Text S1), especially in the case of complex experimental distributions. It is reasonable to hypothesize that strongly non-uniform samples of intervals might affect the shape of the likelihood itself, if only for the simple reason that people practice more on some given intervals. Cognitive, attentional and adaptation mechanisms might play various roles in the interaction between nonuniform priors and likelihoods, in particular without the mitigating effect of knowledge of results. A relatively less explored but important research direction involves extending the model to a biologically more realistic observer and actor model, examining the connections with network dynamics [12], [63] or population coding [31], bridging the gap between a normative description and mechanistic accounts of time perception. Another extension of the model would consider a non-stationary observer, whose response strategy changes from trial to trial (even after training), possibly in order to account for sequential effects of judgement which may be due to an iterative update of the prior [64]–[66]. Finally, whereas our analysis suggests that subjects found it relatively easy to learn unimodal distributions of intervals, bimodal distributions seemed to represent a much harder challenge. Further work is needed to understand human performance and limitations with multimodal temporal distributions.

## Methods

### Ethics statement

The University of Edinburgh School of Informatics ethics committee approved the experimental procedures and all subjects gave informed consent.

### Participants

Twenty-five subjects (17 male and 8 female; age range 19–34 years) including the first author participated in the study. Except for the first author all participants were naïve to the purpose of the study. All participants were right-handed, with normal or corrected-to-normal vision and reported no neurological disorder. Participants were compensated for their time and an additional monetary prize was awarded to the three best naïve performers (lowest mean squared error).

The first author took part in three of the experiments and was included as he represents a highly trained and motivated participant. Therefore it allowed an informal means to assess whether the author's data was different from those of the naïve participants which could reflect a lack of training or motivation. However, analysis of the author's datasets (response biases and moments of the inferred priors) were statistically indistinguishable from the other participants and therefore his data was included in the analysis.

### Materials and stimuli

Participants sat in a dimly lit room, 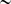
50 cm in front of a Dell M782p CRT monitor (160 Hz refresh rate, 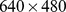 resolution). Participants rested their hand on a high-performance mouse which was fixed to a table and hidden from sight under a cover. The mouse button was sampled at 1 kHz (with a 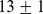 ms latency). Participants wore ear-enclosing headphones (Sennheiser EH2270) playing white noise at a moderate volume, thereby masking any experimental noise. Stimuli were generated by a custom-written program in MATLAB (Mathworks, U.S.A.) using the Psychophysics Toolbox extensions [67], [68]. All timings were calibrated and verified with an oscilloscope.

### Task

Each trial started with the appearance of a grey fixation cross at the center of the screen (27 pixels, 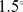 diameter). Participants were required to then click on the mouse button at a time of their choice and this led to a visual flash being displayed on the screen after a delay of 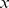 ms which could vary from trial to trial. The flash consisted of a circular yellow dot (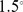 diameter and 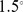 above the fixation cross) which appeared on the screen for 18.5 ms (3 frames). The ‘target’ interval 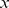 ms was defined from the start of the button press to the first frame of the flash, and was drawn from a block-dependent distribution 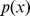. Participants were then required to reproduce the target interval by pressing and holding the mouse button for the same duration. The duration of button press (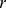 ms) was recorded on each trial. Participants were required to wait at least 250 ms after the flash before starting the interval reproduction, otherwise the trial was discarded and re-presented later. After the button release, 450–850 ms later (uniform distribution), feedback of the performance was displayed for 62 ms. This consisted of a rectangular box (height 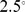, width 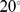) in the lower part of the screen with a central vertical line representing zero error and a dotted line representing the reproduction error on that trial. The horizontal position of the error line relative to the zero-error line was computed as either 
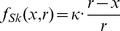
 (Skewed feedback) or 
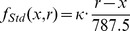
 (Standard feedback), depending on the experimental condition, with 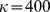 pixels (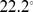). Therefore, for a response 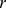 that was shorter than the target interval 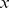 the error line was displayed to the left of the zero-error line, and the converse for a response longer than the target interval. The fixation cross disappeared 500–750 ms after the error feedback, followed by a blank screen for another 500–750 ms and the reappearance of the fixation cross signalled the start of a new trial.

### Experiments

Each session consisted of around 500 trials and was broken up into runs of 84–96 trials. Within each run the number of each interval type was set to reflect the underlying distribution exactly and the order of the presentations was then randomized. However, for the High-Peaked session we ensured that each less likely interval was always preceded by 3–5 ‘peak’ intervals. Subjects could take short breaks between runs.

Each experiment consisted of a number of blocks, each comprising of several sessions. Within each block, the sessions were identical with regard to interval and feedback type. The participants were divided into experimental groups as follows (see also Table 1):

*Experiment 1:* Short Uniform and Long Uniform blocks with Skewed feedback (4 participants, including the first author). *Experiment 2:* Medium Uniform and Medium Peaked blocks with Skewed feedback (6 participants, including the first author). *Experiment 3:* Medium Uniform block with Standard feedback (6 participants, including the first author). *Experiment 4:* Medium High-Peaked block with Standard feedback (3 participants). *Experiment 5:* Medium Bimodal with Standard feedback (4 participants) and Wide Bimodal with Standard feedback (4 participants).

The order of the blocks for Experiments 1 and 2 were randomized across subjects. Each block consisted of three to six sessions, terminating when the participant's performance had stabilized (fractional change in mean squared timing error between sessions less than 0.08). For Experiment 5 we required participants to perform a minimum of five sessions.

### Data analysis

We examined the last two sessions of each block, when performance had plateaued so as to exclude any learning period of the experiment. We analysed all trials for the uniform distributions and Wide Bimodal block. For the non-uniform distributions, we picked a random subset of the frequently-sampled intervals such that all intervals contributed equally in the model comparison (results were mostly independent of the chosen random subset), with the exception of the Wide Bimodal block for which we would have had too few data points per interval. For each subject we therefore analysed about 1000 trials for the Uniform or Wide Bimodal blocks, 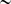
500 for the Peaked or Medium Bimodal block and 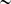
200 trials for the High-Peaked block. We discarded trials with timestamp errors (e.g. multiple or non-detected clicks) and trials whose response durations fell outside a block-dependent allowed window of 225–1237 ms (Short), 300–1462 ms (Medium), 375–1687 ms (Long) and 225–1687 ms (Wide), giving 124 discarded trials out of a total of 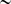
30000 trials (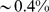). Note that 93% of the discarded trials had response intervals less than 150 ms, which we attribute to accidental mouse presses.

#### Bayesian observer model components

Eqs. 1 and 2 describe the family of Bayesian observers models. The behavior of an observer is defined by the choice of four components:

- a noise model for the sensory estimation process, which can be either constant or scalar:
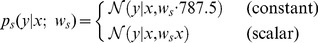
(3)where 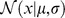 is a normal distribution with mean 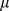 and standard deviation 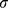.
- a noise model for the motor reproduction process, which can be either constant or scalar:
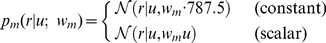
(4)
- the approximation scheme for the priors. We considered: (a) the true, discrete distribution; (b) a single Gaussian with same mean and variance as the true distribution; (c) a mixture of six (ten for the Wide range) 37.5 ms standard deviation Gaussians centered on the true discrete intervals with mixing weights equal to the relative probability of the true intervals; (d) as c but with standard deviation of 75 ms; (e) a continuous uniform distribution from the shortest to the longest interval. For Experiment 2 and 4 we also considered a mixture of two Gaussians with mixing weights 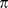 and 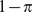, with 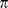 equal to the proportion of ‘peak’ intervals that emerge from the uniform background distribution (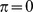 for the Uniform block, 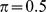 for the Peaked block and 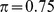 for the High-Peaked block). The first Gaussian is centered on the peak (675 ms) and with a small (f: 37.5 ms) or large (g: 75 ms) standard deviation, the second Gaussian is centered on the mean of the Medium range (787.5 ms) and with standard deviation equal to the discrete Uniform distribution (128.7 ms). Therefore, for the Medium Uniform block approximation schemes f and g reduce to a single Gaussian. Analogously, for Experiment 5 we considered a mixture of three Gaussians with mixing weights 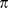, 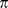 and 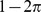, with 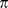 equal to the total frequency of one of the two ‘peaks’ emerging from the uniform background distribution (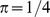 for the Medium Bimodal block and 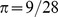 for the Wide Bimodal block). The first two Gaussians are centered on the peaks (Medium: 600 ms and 975 ms; Wide: 525 ms and 1050 ms) and with a small (f: Medium: 37.5 ms; Wide: 61.2 ms) or large (g: twice the small) standard deviation. The third Gaussian is centered on the mean of the range (787.5 ms) and with standard deviation equal to the discrete Uniform distribution over the range (Medium: 128.7 ms; Wide: 251.6 ms). The values of standard deviations for the ‘peak’ Gaussians (small 37.5 ms, large 75 ms) were chosen as 75 ms is the gap between time intervals in all experimental distributions. For the Wide Bimodal block, 61.2 ms is the standard deviation of the sample for three intervals separated by 75 ms.
- the loss function
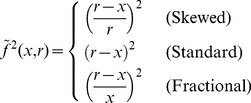
(5)Note that the Fractional error was not used as a feedback shape in the experiments, but we included it as a possibility for the Bayesian observer as it might represent an appropriate error signal if time has a logarithmic representation in the brain [69]. In fact, the logarithmic squared loss reads:
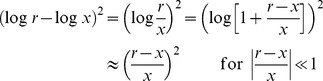
For an analysis with non-quadratic loss function see also Section 2 in Text S1.

#### Bayesian model comparison

For each observer model and each subject's dataset (that is all blocks within an experiment) we calculated the posterior probability of the model given the data, Pr(model| data) 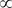 Pr(data| model), assuming a flat prior over the models.

The marginal likelihood is given by
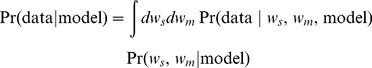
(6)where Pr(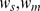
| model) is the prior over the parameters and Pr(data|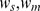, model) is the likelihood of the data given a specific model and value of the parameters. For the prior over the parameters we assumed independence between parameters and models Pr(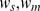
| model) 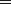 Pr(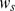)Pr(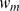) and for both parameters we used a broad Beta prior 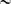 Beta(1.3, 2.6) that slightly favors the range 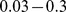 in agreement with a vast literature on human timing errors [34]. The likelihood of the data was computed according to our observer model, Eq. 2, assuming independence across trials:
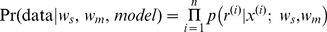
(7)with 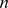 the total number of test trials and 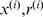 respectively the target interval and response in the 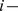
th test trial. Note that the calculation of 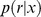 (Eq. 2) requires a computation of the optimal action 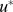, that is, the action 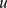 that minimizes the expected loss (Eq. 1). The minimization was performed analytically for the Standard and Fractional loss function and numerically for the Skewed loss function (function fminbnd in MATLAB; we assumed that 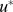 always fell in the interval 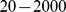 ms; the results were checked against analytical results obtained through a polynomial expansion approximation of the loss function that holds for 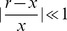).

We computed the marginal likelihood through Eqs. 6 and 7 both with a full numerical integration and using a Laplace approximation (both methods gave identical results). Given the posterior probability for each model, for each subject we calculated the posterior probability for each model component (by fixing a model component and summing over the others); see Table S1. The ‘Bayesian fits’ in Figure 3, 4, 9 top and Figure S5 and S6 in Text S2 were obtained by calculating the model average for the response bias and response standard deviation (the average was taken both over parameters and over models, but typically only one of the models contributed significantly to the integral).

#### Nonparametric reconstruction of the priors

To examine the subjects' priors using a nonparametric approach, for each subject we took the (i) sensory and (ii) motor noise and (iv) loss function, as inferred from the model comparison. We then allowed the priors to vary independently over a broad class of smooth, continuous distributions. For each block, the log prior was specified by the values of ten (14 for the Wide range) control points at 75 ms steps over the ranges: Short 300–1025 ms, Medium 450–1175 ms, Long 600–1325 ms and Wide 300–1325 ms. The control points were centered on the interval range of the block but extended outside the range to allow for tails or shifts. The prior 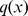 was calculated by interpolating the values of the prior in log space with a Gaussian process [70] with squared exponential covariance function with fixed scale (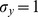 in log space, 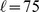 ms) and a small nonzero noise term to favor conditioning. The Gaussian processes were used only as a smooth interpolating method and not as a part of the inference. In order to infer the prior for each subject and block, we sampled from the posterior distribution of priors 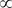 Pr(data| prior, model) using a slice sampling Markov Chain Monte Carlo algorithm [71]. We ran ten parallel chains (3000 burn-in samples, 1500 saved samples per chain) obtaining a total of 15000 sampled priors per subject and block. For each sampled prior we calculated the first four moments (mean, standard deviation, skewness and excess kurtosis) and computed the mean and standard deviation of the moments across the sample sets of individual subjects and over the sample set of all subjects (the latter are shown in Table 2 and 3).

## Supporting Information

Table S1 **Bayesian model comparison: most supported observer model components for Experiments 1–4.** Most supported observer model components (posterior probability), for each subject, according to the Bayesian model comparison.
(PDF)

Text S1 **Additional models and analyses.** This supporting text includes sections on: computation of response bias and standard deviation of the response for the basic Bayesian observer model; a Bayesian observer model with non-quadratic loss function; a Bayesian observer model with lapse; an extended analysis of subjects' sensory and motor variability. Figures S1, S2, S3, S4 are included.
(PDF)

Text S2 **Results** **of Experiments 3 and 4.** Plots of mean response bias and standard deviation of the response for Experiment 3 and 4. Figures S5 and S6 are included.
(PDF)
