# Supplementary material for: Internal Representations of Temporal Statistics and Feedback Calibrate Motor-Sensory Interval Timing
Source: PLoS Comput Biol. 2012 Nov 29;8(11):e1002771. doi: 10.1371/journal.pcbi.1002771 (PMC3510049; doi:10.1371/journal.pcbi.1002771)
Supplement: Table S1 — Bayesian model comparison: most supported observer model components for Experiments 1–4. Most supported observer model components (posterior probability), for each subject, according to the Bayesian model comparison. (PDF) [file pcbi.1002771.s001.pdf]

Subject	Sensory likelihood		Motor likelihood		Subjective prior		Loss function	
				**Experiment 1**				
LA	Sc	(1.000)	Sc	(1.000)	b	(1.000)	Sk	(1.000)
JW	Sc	(0.967)	Sc	(1.000)	b	(0.960)	St	(1.000)
TL	Cn	(1.000)	Sc	(1.000)	b	(1.000)	Sk	(1.000)
DB	Sc	(1.000)	Sc	(0.974)	e	(0.997)	St	(1.000)
				**Experiment 2**				
LA	Sc	(1.000)	Sc	(0.997)	g	(1.000)	Sk	(1.000)
AC	Cn	(1.000)	Sc	(1.000)	f	(0.978)	St	(1.000)
AP	Cn	(1.000)	Sc	(0.981)	b	(1.000)	Sk	(1.000)
HH	Sc	(0.997)	Sc	(0.997)	g	(0.998)	Sk	(0.875)
JB	Sc	(0.998)	Cn	(0.996)	f	(0.997)	Sk	(1.000)
TZ	Sc	(1.000)	Cn	(1.000)	d	(0.976)	Sk	(1.000)
				**Experiment 3**				
LA	Cn	(0.910)	Sc	(0.990)	b	(1.000)	St	(0.993)
NY	Sc	(0.988)	Sc	(0.780)	b	(1.000)	Fr	(1.000)
JL	Sc	(0.528)	Sc	(1.000)	b	(0.999)	St	(1.000)
RD	Cn	(1.000)	Sc	(0.996)	b	(0.998)	Fr	(1.000)
PD	Sc	(0.758)	Cn	(1.000)	b	(0.999)	St	(1.000)
JE	Cn	(0.896)	Sc	(0.912)	b	(1.000)	St	(1.000)
				**Experiment 4**				
RR	Cn	(0.986)	Sc	(0.950)	a	(0.998)	St	(−)
DD	Cn	(0.726)	Cn	(0.641)	f	(0.511)	St	(−)
					g	(0.486)		
NG	Cn	(0.980)	Sc	(0.973)	b	(0.503)	St	(−)
					g	(0.458)		

Table S1. Bayesian model comparison: most supported observer model components for Experiments 1–4. Most supported observer model components (posterior probability), for each subject, according to the Bayesian model comparison. A posterior probability $p > 0.95$ should be considered suggestive evidence, and $p > 0.99$ significant (posterior probability $p > 0.9995$ is written as 1.000, with a slight abuse of notation). The sensory and motor likelihoods can either be constant (Cn) or scalar (Sc); the subjective priors (a-g) are described in the Methods section (see main text); the loss function can be Skewed (Sk), Standard (St) or Fractional (Fr) (see also Figure 6 in main text). Note the switch in preferred loss function from Experiments 1 and 2 (which received Skewed feedback) to Experiment 3 (which received Standard feedback). In Experiment 4 the loss function was fixed to Standard to constrain the model selection.
