# Supplementary material for: Internal Representations of Temporal Statistics and Feedback Calibrate Motor-Sensory Interval Timing
Source: PLoS Comput Biol. 2012 Nov 29;8(11):e1002771. doi: 10.1371/journal.pcbi.1002771 (PMC3510049; doi:10.1371/journal.pcbi.1002771)
Supplement: Text S1 — Additional models and analyses. This supporting text includes sections on: computation of response bias and standard deviation of the response for the basic Bayesian observer model; a Bayesian observer model with non-quadratic loss function; a Bayesian observer model with lapse; an extended analysis of subjects' sensory and motor variability. Figures S1, S2, S3, S4 are included. (PDF) [file pcbi.1002771.s002.pdf]

Internal Representations of Temporal Statistics and Feedback Calibrate Motor-Sensory Interval Timing

L. Acerbi, D. M. Wolpert and S. Vijayakumar

Supporting Text S1 – Additional models and analyses

Contents

1 Computation of response bias and standard deviation of the response 2

2 Non-quadratic loss function 4

3 Bayesian observer model with lapse 4

4 Sensory and motor variability 5

4.1 Measuring sensory and motor noise 6

4.2 Internal knowledge of estimation variability 7

4.3 Generalized law for motor noise 8

1 Computation of response bias and standard deviation of the response

The key equations of our model are Eqs. 1 (optimal action $u^*(y)$ for internal measurement y) and 2 (probability of response r given the true time interval x); see main text. In particular, they allow us to compute the response bias and standard deviation (sd) which are shown in the plots.

As intermediate calculations, the mean response for interval x is

$$\mathbb{E}\left[r\right]_{p(r|x)} = \int p(r|x) r \, dr = \int \left[\int p_s(y|x) p_m(r|u^*(y)) \, dy \right] r \, dr = \int p_s(y|x) u^*(y) \, dy \tag{S1}$$

and, analogously, the the second moment of the response reads

$$\begin{aligned} \mathbb{E}\left[r^2\right]_{p(r|x)} &= \int p(r|x) r^2 \, dr = \int \left[\int p_s(y|x) p_m(r|u^*(y)) \, dy \right] r^2 \, dr \\ &= \int p_s(y|x) \left[u^*(y)^2 + \sigma_m^2(u^*(y)) \right] \, dy. \end{aligned} \tag{S2}$$

In the above derivations we have used the fact that the motor likelihood $p_m(r|u)$ is modelled as a Gaussian with mean u and variance $\sigma_m^2(u)$ (the specific function for the variance depends on the observer model; see Methods).

From Eqs. S1 and S2 we can compute

$$\text{Reponse bias } (x) = \mathbb{E}\left[r\right]_{p(r|x)} - x, \qquad \text{Response sd } (x) = \sqrt{\mathbb{E}\left[r^2\right]_{p(r|x)} - \left(\mathbb{E}\left[r\right]_{p(r|x)} \right)^2}. \tag{S3}$$

Note that the optimal action $u^*(y)$ is a key element of all equations. In particular, the mean response in Eq. S1 is obtained by convolving the optimal action with the sensory likelihood. In other words, plots of the mean response are smoothed versions of plots of the optimal action; the same relationship holds for the response bias and shifted optimal action $u^*(y) - y$ (see Figure S1).

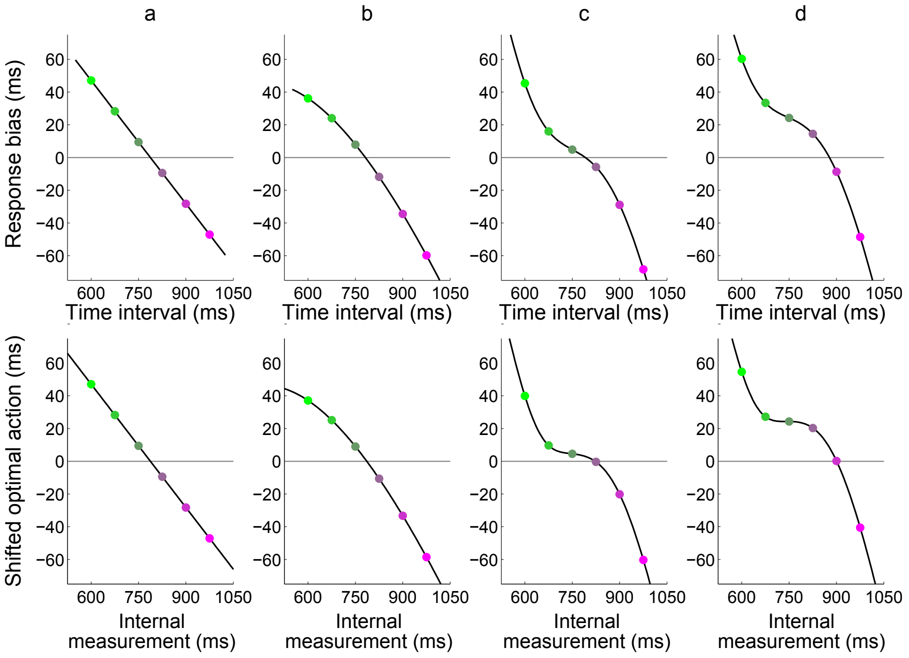

Figure S1. Comparison of response bias and (shifted) optimal action. Response bias and (shifted) optimal action for four different ideal observers (*columns* **a-d**) are shown (see Figure 1 in main text). **Top:** Response bias for the example observer models taken from Figure 1 in the paper. **Bottom:** Shifted optimal action $u^*(y) - y$ for the same models. For ease of comparison, different colored dots mark a discrete set of interval durations. Note the similarity between the two rows; the mean response is in fact obtained by convolving the optimal action with the sensory noise (Eq. S1).

2 Non-quadratic loss function

Our basic model assumed a quadratic (or pseudo-quadratic) loss function that was obtained by squaring the subjective error map $\widetilde{f}(x, r)$ (Eq. 1 in main text). The exponent 2 allowed a semi-analytical solution of Eq. 1, which made tractable the problems of (a) computing the marginal likelihood for a relatively large class of models and (b) nonparametrically inferring the subjects' priors (see main text). However, previous work has shown that people in sensorimotor tasks may be instead following a sub-quadratic loss function [1].

For the sake of completeness, we explored an extended model with non-quadratic loss functions. For computational reasons we could not perform a full Bayesian model comparison, but we considered only the 'best' observer model per subject. We used datasets from Experiments 1 and 2 as they comprised two distinct blocks per subject, which provided more data points and reduced risk of overfitting. For each subject we chose the most supported model components for the sensory and motor likelihood and the shape of the subjective error mapping (Standard, Skewed or Fractional), whereas for the prior we took the nonparametrically inferred priors. However, the exponent of the loss function was now free to vary, so that the equation for the optimal action reads

$$u^*(y) = \arg\min_u \int p_s(y|x; w_s) q(x) p_m(r|u; w_m) \left| \widetilde{f}(x, r) \right|^\kappa dx\, dr. \tag{S4}$$

where $\kappa > 0$ is a continuous free parameter representing the exponent of the loss function. Eq. S4 was solved numerically (function `fminbnd` and `trapz` in MATLAB) for various values of y and then interpolated. Through Eqs. 2 and 7 we computed for each subject the posterior probability of the exponent $\Pr(\kappa|\text{data}) \propto \Pr(\text{data}|\kappa)\Pr(\kappa)$, where we assumed an (improper) uniform prior on κ.

Results are shown in Figure S2 as a box plot for each subject's inferred κ. Taking the median of the posterior distribution as the inferred value for κ, the exponent averaged across subjects (excluding one outlier) is 1.88 ± 0.06 which is marginally lower than 2 (one-sample t-test $p < 0.07$). (Taking the mean of the posterior instead of the median renders analogous results.) This result is in qualitative agreement with [1] which found that subjects were following a sub-quadratic loss function (with exponent 1.72 ± 0.03 for a power law). Our average inferred exponent is however higher, and only marginally lower than 2, but this might be due to the fact that the subjects' priors have been inferred under the assumption of a quadratic loss function, and therefore priors may be already 'fitting' some features of the data that were due instead to a sub-quadratic loss function. The structure of the model does not currently allow for a simultaneous inference of both nonparametric priors and exponent of the loss function computationally, which is an open problem for future work.

3 Bayesian observer model with lapse

We extended the Bayesian observer model described in the paper (Eqs. 1 and 2) by introducing for each subject in Experiments 1 and 2 a third continuous parameter, the *probability of lapse* λ. For each trial, the observer has some probability λ of ignoring the current stimulus and responding with uniform probability over the range of allowed responses – a very simple model of data outliers due to subjects' errors. The response probability with lapse reads

$$p_{\text{lapse}}(r|x; w_s, w_m, \lambda) = \lambda \frac{1}{L} + (1-\lambda) p(r|x; w_s, w_m) \tag{S5}$$

where L is the allowed response window duration (which is block-dependent, see Data Analysis). By using Eq. S5 in Eq. 7 (see Methods) we computed the marginal likelihood of models with lapse, extracted the most supported model components and hence inferred the subjective priors.

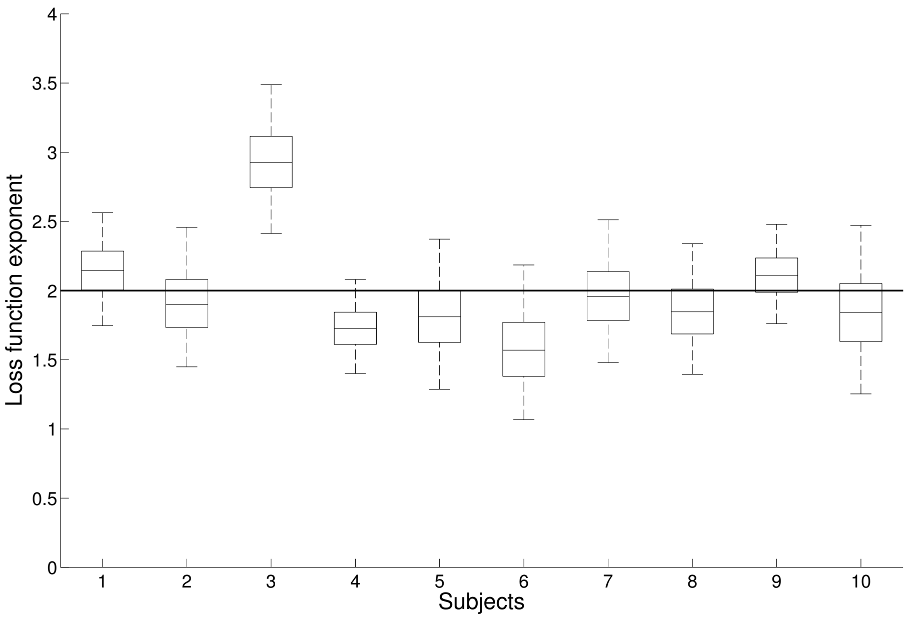

Figure S2. Non-quadratic loss function. Inferred exponents of the loss function for subjects in Experiment 1 (ss $1 - 4$) and 2 (ss $5 - 10$). The box plots have lines at the lower quartile, median, and upper quartile values; whiskers cover 95% confidence interval. Excluding one outlier (s 3), the average inferred exponent is marginally lower than 2 ($p < 0.07$).

The average moments of the reconstructed priors did not differ significantly from the ones computed with the basic model without lapse (see Table 2), and in particular the kurtosis was similar, being in general systematically higher than the true distribution kurtosis. The excess kurtosis for the observers with lapse, computed by averaging the moments of sampled priors pooled from all subjects, was (mean ± 1 s.d.): 0.85 ± 1.30 (Short Uniform), 0.70 ± 1.01 (Long Uniform); 0.91 ± 1.57 (Medium Uniform), 1.87 ± 1.84 (Medium Peaked); as opposed to a true excess kurtosis of -1.27 (Uniform blocks) and 0.09 (Peaked block).

4 Sensory and motor variability

The sensory (estimation) and motor (reproduction) likelihoods in our observer's model were represented by normal distributions whose standard deviation (either constant or 'scalar', Figure 6 i and ii, see paper) was governed by the two parameters w_s, w_m, respectively for the sensory and motor component. We describe here a set of additional experiments and analyses which tested various hypotheses about our subjects' sensorimotor likelihoods.

First of all, we examined whether the parameter values w_s, w_m inferred from the data corresponded to direct measures of sensory and motor variability gathered in different tasks. We found a good agreement at the group level for both parameters and a good correlation for the individual values of the sensory noise (see 'Measuring sensory and motor noise').

With an additional model comparison, we checked whether, according to our data, subjects' 'knew' their own sensory (estimation) variability; that is, we examined whether their internal estimate of their sensory variability matched their objective sensory variability (both quantities were computed from the model). The analysis suggests that subjects were generally 'aware' of their own sensory variability (refer

to subsection below on 'Internal knowledge of estimation variability'). We did not perform an analogous study on the motor variability as the problem becomes under-constrained (see below).

At last, to see whether we could better understand the form of the motor noise we analyzed our data with a 'generalized' model with 2 parameters governing the growth of the standard deviation of the motor noise. Interestingly, the generalized model did not perform better in terms of model comparison than the 1-parameter scalar model (refer to subsection below on 'Generalized law for motor noise').

4.1 Measuring sensory and motor noise

For each subject in Experiments 1 and 2 we computed the posterior distribution of w_s, w_m (weighted average over all models) and took the mean of the posterior as the 'model-inferred' sensory and motor variability. We examined whether the model-inferred values corresponded to direct measures of sensory and motor variability (w'_s, w'_m) obtained through additional experiments. We directly measured each subject's sensory variability w'_s in a two-alternative forced choice time interval discrimination task, and analogously we directly measured the subjects' motor variability w'_m in a time interval 'production' task (see below, Methods, for details).

The comparison between the model-inferred values and the directly-measured ones is shown in Figure S3 for the sensory (left) and motor (right) noise parameters. For sensory variability, we found that w'_s had a good correlation ($R^2 = 0.77$) with w_s, and the group means were in good agreement ($\overline{w_s} = 0.157 \pm 0.002$, $\overline{w'_s} = 0.166 \pm 0.009$). For the motor variability, the group means were quantitatively similar, even though in slight statistical disagreement ($\overline{w_m} = 0.072 \pm 0.001$, $\overline{w'_m} = 0.078 \pm 0.001$), but we did not find a correlation between w'_m and w_m (see Discussion). These results suggest that the model parameters for the 'noise properties' extracted from the full model were in agreement with independent measures of these noise properties in isolation. Interestingly, independent measurements of the sensory noise had predictive power on the subjects' performance even at the individual level (data not shown), due to the good correlation with the sensory model parameter.

The lack of correlation for the motor noise parameter at the individual level may have been due to other noise factors, not contemplated in the model, that influenced the variance of the produced response (e.g. noise in the decision making process, non-Gaussian likelihoods, deviations from the exact scalar property, etc.).

Methods

Each participant of Experiments 1 and 2 took part in a side sensory and motor measurement session. In these sessions all stimuli and materials were identical to the ones presented in the main experiment (see Methods in main text); the design of these experiments itself was chosen to be as similar as possible to the main experiment, but focussing only on the sensory (estimation) or motor (reproduction) part of the task.

In the sensory noise measurement session, $\sim$ 320 trials, in each trial subjects clicked on a mouse button and a dot flashed on screen after a given duration (x_1 ms). Subjects clicked again on the mouse button, and a second dot flashed on screen after x_2 ms. At the end of each trial subjects had to specify which interval was longer through a two-alternative forced choice. Correct responses received a tone as positive feedback. Intervals x_1 and x_2 were adaptively chosen from the range 300–1275 ms on a trial by trial basis in order to approximately maximize the expected gain in information about the sensory variability of the subject (we adapted the algorithm described in [2]).

In the motor noise measurement session, each trial subjects had to reproduce a given block-dependent interval by holding the mouse button. Subjects received visual feedback of their performance through the Skewed error mapping (as in Experiments 1 and 2). For each block the target interval was always the same (500, 750 or 1000 ms) and the subjects were instructed about it. Subjects performed on the

three intervals twice, in a randomized order, for a total of six blocks (30 trials per block, the first five trials were discarded).

For each subject we built simple ideal observer models of the interval discrimination and interval reproduction tasks in which the sensory and motor variability could either be constant or scalar (according to the results of the model comparison in the main experiment). We computed the posterior distributions of the sensory and motor noise parameters, and took the mean of the posterior as the 'directly-measured' noise parameters (w'_s, w'_m).

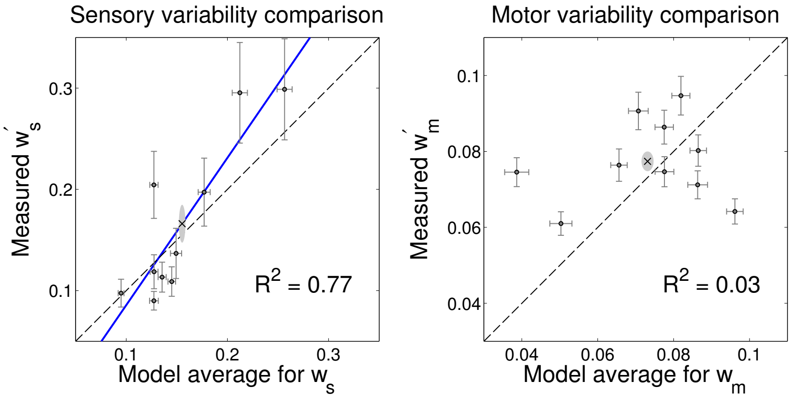

Figure S3. Comparison of sensory and motor noise parameters (main experiment vs direct measurements). For each participant of the main experiments (Experiment 1 and 2, $n = 10$) we independently measured the sensory (w'_s) and motor (w'_m) variabilities in a time-interval discrimination session and a time interval reproduction session with performance feedback (see text for details). We built simple probabilistic models for the above tasks and computed the posterior mean and standard deviation for w'_s and w'_m. For each subject we also calculated the posterior mean and standard deviation for the parameters w_s and w_m that appear in our Bayesian ideal observer model, averaged over all models (weighted by the model posterior probability). Ideally, the couples of parameters (w_s, w'_s) and (w_m, w'_m) reflected the same objective features of the subjects measured in distinct, indepedent tasks. The parameters are compared in the figure, (w_s, w'_s) to the left and (w_m, w'_m) to the right, each circle is a participant's parameters mean ±1 s.d. We also plotted the group mean (crosses, shaded area 95% confidence interval). The group means are $\overline{w_s} = 0.157 \pm 0.002$, $\overline{w'_s} = 0.166 \pm 0.009$; $\overline{w_m} = 0.072 \pm 0.001$, $\overline{w'_m} = 0.078 \pm 0.001$.

4.2 Internal knowledge of estimation variability

Our modelling framework allowed us to ask whether subjects 'knew' their own sensory (estimation) variability in the task [3–5]. We extended our original model by introducing a distinction between the objective sensory variability w_s and the subjective estimate the Bayesian observer had of its value, $\widetilde{w}_s$. The computation of the optimal action was modified accordingly,

$$u^*(y) = \arg\min_u \int p_s(y|x; \widetilde{w}_s) q(x) p_m(r|u; w_m) \widetilde{f}^2(x, r)\, dx\, dr \tag{S6}$$

which is identical to Eq. 1 but note that the expected loss depends now on the subjective value $\widetilde{w}_s$ instead of w_s. The other equations of the model remained unchanged as they depend on the objective sensory noise.

We performed a full Bayesian model comparison with the extended model, where all components (likelihoods, prior, loss function) were free to vary as per the basic model comparison (see paper); the only difference being the presence of three continuous parameters $(w_s, w_m, \widetilde{w}_s)$ and Eq. S6. We limited our analysis to Experiment 1 and 2, as they had two distinct blocks per subject and therefore more data and reduced ambiguity and risk of overfitting. Results of the model comparison showed that the extended models did not gain a significant advantage in terms of marginal likelihood (data not shown), i.e. the distinction between objective and subjective sensory variability did not appear to be a relevant feature in explaining our data. This result suggests that most subjects had a reasonably accurate estimate of their own sensory variability.

Note that an analogous study for the motor (reproduction) variability is not feasible with our dataset as the problem becomes in this case under-constrained. In fact, if we separate the objective motor variability w_m from its subjective estimate $\widetilde{w}_m$, some observer models do not even depend on $\widetilde{w}_m$ (e.g. an observer with constant motor likelihood and Standard loss function), and others show only a weak dependence. In order to meaningfully test whether people 'knew' their own motor variability a much stronger asymmetry in the loss function is needed, along with some other experimental manipulations (see for instance [3]).

4.3 Generalized law for motor noise

A recent study has shown violations of the scalar property for motor timing [6]; see also [7] for a review. The scalar property, taken literally, entails that motor variability decreases to zero for vanishing time intervals, which is quite unlikely; a more realistic assumption is that motor noise must reach a lower bound. The fact that many studies in time interval reproduction have shown a good agreement with the scalar property may simply mean that the lower bound was negligible for the considered interval ranges.

To verify whether this is the case for our work, we considered a 2-parameters model for the motor variability which consists of two independent noise sources, one of which is constant and the other which follows the scalar property. In this model, the equation for the motor variance is

$$\sigma_m^2(u) = \sigma_0^2 + w_m^2 u^2 \qquad \text{(generalized motor variability)} \qquad \text{(S7)}$$

where u is the desired reproduction interval, w_m is the scalar coefficient (Weber's fraction) and σ_0 represents the lower bound for the motor noise.

We ran a full Bayesian model comparison on all datasets (including the new ones), adding the 'generalized' motor variability as a possible choice for the motor likelihood component, in addition to the basic constant and scalar motor components considered before. All other components (sensory likelihood, prior, loss function) were free to vary as per the basic model comparison (see paper).

We found that observer models with generalized motor variability obtained slightly better fits in some cases (Figure S4), but they performed better in terms of marginal likelihood (with respect to the scalar or constant models) only for two subjects in Experiment 1. For all remaining subjects and experiments the extended model did not represent an improvement in marginal likelihood – that is, the minimal gain in model fitting was hampered by the 'cost' of the additional parameter σ_0, meaning that in general the model does not represent a better explanation for the data.

It is not surprising that the subjects who gained some benefit from the addition of the constant noise term belonged to Experiment 1, since this experiment included a Short block and therefore might be more sensitive to the presence of a constant error for short intervals. These results show that while Eq. S7 probably applies to small intervals [6], it seems that in our study the lower bound σ_0 is not relevant for explaining the data and can therefore be ignored with a good approximation.

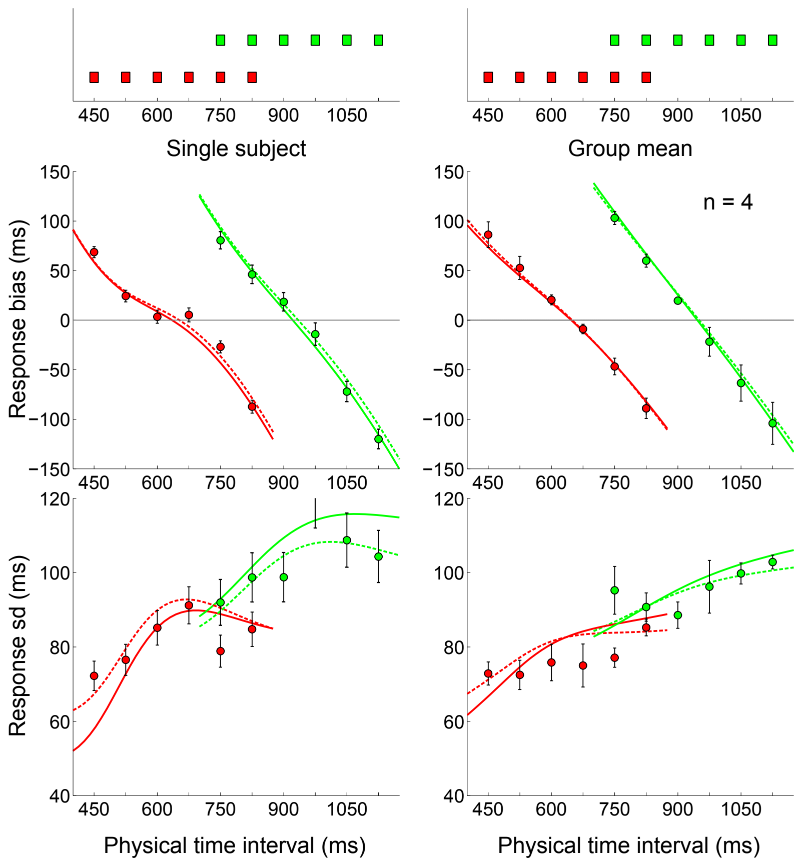

Figure S4. Experiment 1: comparison between basic models and models with generalized motor variability. *Very top:* Experimental distributions for Short Uniform (red) and Long Uniform (green) blocks, repeated on top of both columns. *Left column:* Mean response bias (average difference between the response and true interval duration, top) and standard deviation of the response (bottom) for a representative subject in both blocks (red: Short Uniform; green: Long Uniform). Error bars denote s.e.m. Continuous lines represent the Bayesian model 'fit' obtained averaging the predictions of the most supported basic model components (scalar or constant); dashed lines are model fits which include the generalized motor variability in the model comparison. The subject shown is the one who gained the most by the introduction of the general linear motor likelihood. *Right column:* Mean response bias (top) and standard deviation of the response (bottom) across subjects in both blocks (mean ± s.e.m. across subjects). Continuous lines represent the Bayesian model 'fit' obtained averaging the predictions of the most supported basic models across subjects; dashed lines are model fits which include the generalized motor variability. Although providing slightly better fits, the extended model did not represent a substantial improvement over the 1-parameter motor noise models.

References

1. Körding KP, Wolpert DM (2004) The loss function of sensorimotor learning. Proc Natl Acad Sci U S A 101: 9839–9842.

2. Kontsevich LL, Tyler CW (1999) Bayesian adaptive estimation of psychometric slope and threshold.

Vision Res 39: 2729–37.

3. Hudson T, Maloney L, Landy M (2008) Optimal compensation for temporal uncertainty in movement planning. PLoS Comput Biol 4: e1000130.

4. Barthelmé S, Mamassian P (2009) Evaluation of objective uncertainty in the visual system. PLoS Comput Biol 5: e1000504.

5. Battaglia PW, Kersten D, Schrater PR (2011) How haptic size sensations improve distance perception. PLoS Comput Biol 7: e1002080.

6. Laje R, Cheng K, Buonomano D (2011) Learning of temporal motor patterns: an analysis of continuous versus reset timing. Front Integr Neurosci 5.

7. Lewis PA, Miall RC (2009) The precision of temporal judgement: milliseconds, many minutes, and beyond. Proc Biol Sci 364: 1897–1905.
