# Supplementary material for: Internal Representations of Temporal Statistics and Feedback Calibrate Motor-Sensory Interval Timing
Source: PLoS Comput Biol. 2012 Nov 29;8(11):e1002771. doi: 10.1371/journal.pcbi.1002771 (PMC3510049; doi:10.1371/journal.pcbi.1002771)
Supplement: Text S2 — Results of Experiments 3 and 4. Plots of mean response bias and standard deviation of the response for Experiment 3 and 4. Figures S5 and S6 are included. (PDF) [file pcbi.1002771.s003.pdf]

Internal Representations of Temporal Statistics and Feedback Calibrate Motor-Sensory Interval Timing

L. Acerbi, D. M. Wolpert and S. Vijayakumar

Supporting Text S2 – Results of Experiments 3 and 4

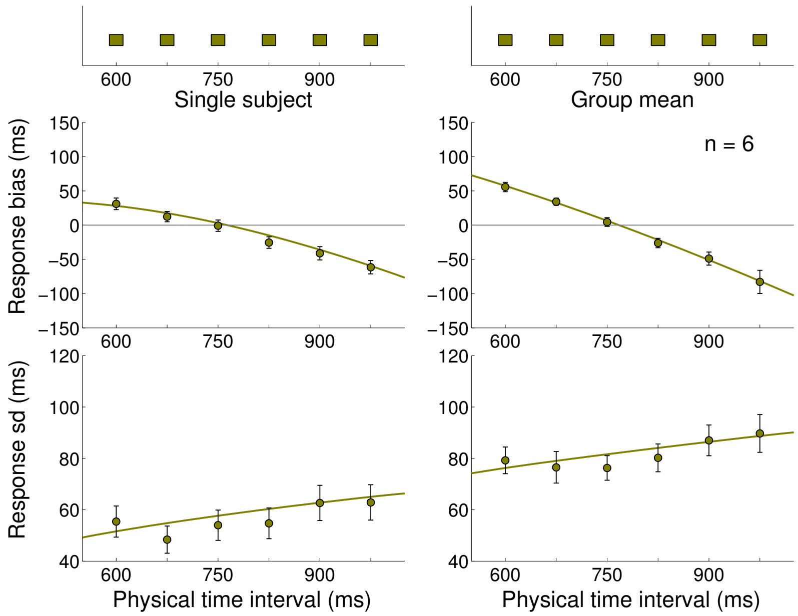

Figure S5. Experiment 3: Medium Uniform block with Standard feedback. *Very top:* Experimental distribution for Medium Uniform block, repeated on top of both columns. *Left column:* Mean response bias (average difference between the response and true interval duration, top) and standard deviation of the response (bottom) for a representative subject. Error bars denote s.e.m. Continuous lines represent the Bayesian model 'fit' obtained averaging the predictions of the most supported models (Bayesian model averaging). *Right column:* Mean response bias (top) and standard deviation of the response (bottom) across subjects (mean ± s.e.m. across subjects). Continuous lines represent the Bayesian model 'fit' obtained averaging the predictions of the most supported models across subjects.

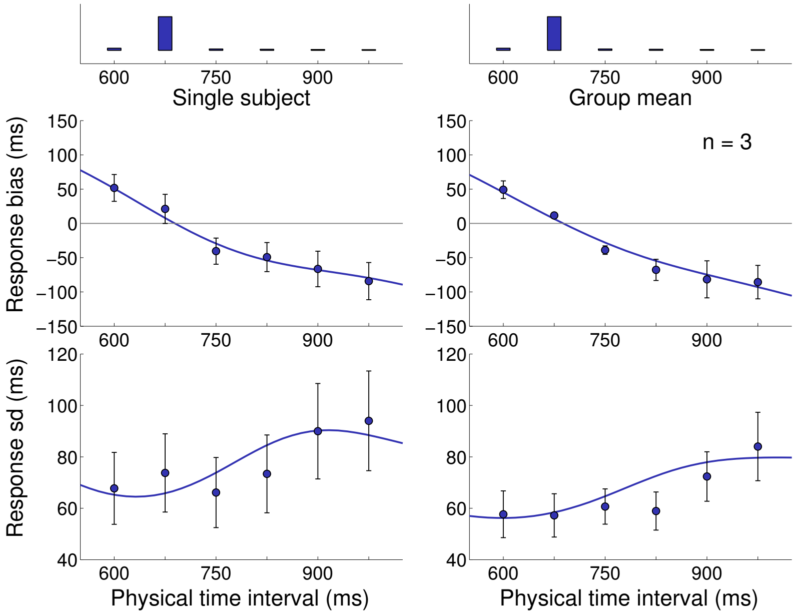

Figure S6. Experiment 4: Medium High-Peaked block. *Very top:* Experimental distribution for Medium High-Peaked block, repeated on top of both columns. *Left column:* Mean response bias (average difference between the response and true interval duration, top) and standard deviation of the response (bottom) for a representative subject. Error bars denote s.e.m. Continuous lines represent the Bayesian model 'fit' obtained averaging the predictions of the most supported models (Bayesian model averaging). *Right column:* Mean response bias (top) and standard deviation of the response (bottom) across subjects (mean $\pm$ s.e.m. across subjects). Continuous lines represent the Bayesian model 'fit' obtained averaging the predictions of the most supported models across subjects.
